# Differences Between Plasma and Cerebrospinal Fluid Glial Fibrillary Acidic Protein Levels Across the Alzheimer Disease Continuum

**DOI:** 10.1001/jamaneurol.2021.3671

**Published:** 2021-10-18

**Authors:** Andréa L. Benedet, Marta Milà-Alomà, Agathe Vrillon, Nicholas J. Ashton, Tharick A. Pascoal, Firoza Lussier, Thomas K. Karikari, Claire Hourregue, Emmanuel Cognat, Julien Dumurgier, Jenna Stevenson, Nesrine Rahmouni, Vanessa Pallen, Nina M. Poltronetti, Gemma Salvadó, Mahnaz Shekari, Gregory Operto, Juan Domingo Gispert, Carolina Minguillon, Karine Fauria, Gwendlyn Kollmorgen, Ivonne Suridjan, Eduardo R. Zimmer, Henrik Zetterberg, José Luis Molinuevo, Claire Paquet, Pedro Rosa-Neto, Kaj Blennow, Marc Suárez-Calvet

**Affiliations:** 1 Institute of Neuroscience and Physiology, Department of Psychiatry and Neurochemistry, The Sahlgrenska Academy, University of Gothenburg, Gothenburg, Sweden; 2 Translational Neuroimaging Laboratory, McGill Centre for Studies in Aging, McGill University, Montreal, Quebec, Canada; 3 Barcelonaßeta Brain Research Center, Pasqual Maragall Foundation, Barcelona, Spain; 4 IMIM (Hospital del Mar Medical Research Institute), Barcelona, Spain; 5 Centro de Investigación Biomédica en Red de Fragilidad y Envejecimiento Saludable (CIBERFES), Madrid, Spain; 6 Universitat Pompeu Fabra, Barcelona, Spain; 7 Université de Paris, Institut national de la santé et de la recherche médicale U1144 Optimisation Thérapeutique en Neuropsychopharmacologie, Paris, France; 8 Centre de Neurologie Cognitive, Groupe Hospitalo Universitaire Assistance Publique Hôpitaux de Paris Nord Hôpital Lariboisière Fernand-Widal, Paris, France; 9 Wallenberg Centre for Molecular and Translational Medicine, Department of Psychiatry and Neurochemistry, Institute of Neuroscience and Physiology, The Sahlgrenska Academy, University of Gothenburg, Gothenburg, Sweden; 10 Maurice Wohl Clinical Neuroscience Institute, Institute of Psychiatry, Psychology and Neuroscience, King’s College London, London, United Kingdom; 11 National Institute for Health Research Biomedical Research Centre for Mental Health and Biomedical Research Unit for Dementia at South London and Maudsley National Health Service Foundation, London, United Kingdom; 12 Centro de Investigación Biomédica en Red Bioingeniería, Biomateriales y Nanomedicina, Madrid, Spain; 13 Roche Diagnostics GmbH, Penzberg, Germany; 14 Roche Diagnostics International Ltd, Rotkreuz, Switzerland; 15 Department of Pharmacology, Graduate Program in Biological Sciences: Biochemistry (PPGBioq) and Phamacology and Therapeutics (PPGFT), Universidade Federal do Rio Grande do Sul, Porto Alegre, Brazil; 16 Clinical Neurochemistry Laboratory, Sahlgrenska University Hospital, Mölndal, Sweden; 17 Department of Neurodegenerative Disease, University College London Institute of Neurology, London, United Kingdom; 18 UK Dementia Research Institute at University College London, London, United Kingdom; 19 Montreal Neurological Institute, Montreal, Quebec, Canada; 20 Department of Neurology and Neurosurgery, McGill University, Montreal, Quebec, Canada; 21 Servei de Neurologia, Hospital del Mar, Barcelona, Spain

## Abstract

**Importance:**

Glial fibrillary acidic protein (GFAP) is a marker of reactive astrogliosis that increases in the cerebrospinal fluid (CSF) and blood of individuals with Alzheimer disease (AD). However, it is not known whether there are differences in blood GFAP levels across the entire AD continuum and whether its performance is similar to that of CSF GFAP.

**Objective:**

To evaluate plasma GFAP levels throughout the entire AD continuum, from preclinical AD to AD dementia, compared with CSF GFAP.

**Design, Setting, and Participants:**

This observational, cross-sectional study collected data from July 29, 2014, to January 31, 2020, from 3 centers. The Translational Biomarkers in Aging and Dementia (TRIAD) cohort (Montreal, Canada) included individuals in the entire AD continuum. Results were confirmed in the Alzheimer’s and Families (ALFA+) study (Barcelona, Spain), which included individuals with preclinical AD, and the BioCogBank Paris Lariboisière cohort (Paris, France), which included individuals with symptomatic AD.

**Main Outcomes and Measures:**

Plasma and CSF GFAP levels measured with a Simoa assay were the main outcome. Other measurements included levels of CSF amyloid-β 42/40 (Aβ42/40), phosphorylated tau181 (p-tau181), neurofilament light (NfL), Chitinase-3-like protein 1 (YKL40), and soluble triggering receptor expressed on myeloid cells 2 (sTREM2) and levels of plasma p-tau181 and NfL. Results of amyloid positron emission tomography (PET) were available in TRIAD and ALFA+, and results of tau PET were available in TRIAD.

**Results:**

A total of 300 TRIAD participants (177 women [59.0%]; mean [SD] age, 64.6 [17.6] years), 384 ALFA+ participants (234 women [60.9%]; mean [SD] age, 61.1 [4.7] years), and 187 BioCogBank Paris Lariboisière participants (116 women [62.0%]; mean [SD] age, 69.9 [9.2] years) were included. Plasma GFAP levels were significantly higher in individuals with preclinical AD in comparison with cognitively unimpaired (CU) Aβ-negative individuals (TRIAD: Aβ-negative mean [SD], 185.1 [93.5] pg/mL, Aβ-positive mean [SD], 285.0 [142.6] pg/mL; ALFA+: Aβ-negative mean [SD], 121.9 [42.4] pg/mL, Aβ-positive mean [SD], 169.9 [78.5] pg/mL). Plasma GFAP levels were also higher among individuals in symptomatic stages of the AD continuum (TRIAD: CU Aβ-positive mean [SD], 285.0 [142.6] pg/mL, mild cognitive impairment [MCI] Aβ-positive mean [SD], 332.5 [153.6] pg/mL; AD mean [SD], 388.1 [152.8] pg/mL vs CU Aβ-negative mean [SD], 185.1 [93.5] pg/mL; Paris: MCI Aβ-positive, mean [SD], 368.6 [158.5] pg/mL; AD dementia, mean [SD], 376.4 [179.6] pg/mL vs CU Aβ-negative mean [SD], 161.2 [67.1] pg/mL). Plasma GFAP magnitude changes were consistently higher than those of CSF GFAP. Plasma GFAP more accurately discriminated Aβ-positive from Aβ-negative individuals than CSF GFAP (area under the curve for plasma GFAP, 0.69-0.86; area under the curve for CSF GFAP, 0.59-0.76). Moreover, plasma GFAP levels were positively associated with tau pathology only among individuals with concomitant Aβ pathology.

**Conclusions and Relevance:**

This study suggests that plasma GFAP is a sensitive biomarker for detecting and tracking reactive astrogliosis and Aβ pathology even among individuals in the early stages of AD.

## Key Points

**Question:**

What are the levels of plasma glial fibrillary acidic protein (GFAP) throughout the Alzheimer disease (AD) continuum, and how do they compare with the levels of cerebrospinal fluid (CSF) GFAP?

**Findings:**

In this cross-sectional study, plasma GFAP levels were elevated in the preclinical and symptomatic stages of AD, with levels higher than those of CSF GFAP. Plasma GFAP had a higher accuracy than CSF GFAP to discriminate between amyloid-β (Aβ)–positive and Aβ-negative individuals, also at the preclinical stage.

**Meaning:**

This study suggests that plasma GFAP is a sensitive biomarker that significantly outperforms CSF GFAP in indicating Aβ pathology in the early stages of AD.

## Teaser

This cross-sectional cohort study evaluates plasma glial fibrillary acidic protein levels throughout the entire Alzheimer disease continuum, from preclinical Alzheimer disease to Alzheimer disease dementia, compared with cerebrospinal fluid glial fibrillary acidic protein.

## Introduction

The rapid advancements in the development of blood biomarkers to accurately detect Alzheimer disease (AD) point to a prompt application of these biomarkers in clinical routine and clinical trials. This application is especially true for individuals with preclinical AD, as scalable and less invasive biomarkers are needed to screen large populations of cognitively unimpaired (CU) individuals to test innovative interventions.

Currently, the most promising blood biomarkers for detecting AD are the phosphorylated tau species (p-tau)^1,2,3,4,5,6^ and amyloid-β 42/40 (Aβ42/40) ratio.^7,8,9,10,11,12^ However, it is still desirable to have more sensitive blood biomarkers for preclinical AD. Alzheimer disease pathology is associated with morphologic, molecular, and functional remodeling of astrocytes, a process termed *reactive astrogliosis*.^13,14^ However, few astrocyte imaging and fluid biomarkers have been investigated.^15^ Blood levels of glial fibrillary acidic protein (GFAP), a reactive astrogliosis biomarker, are higher in individuals with preclinical AD, constituting a promising candidate biomarker for this early stage of the disease.^16^ A recent meta-analysis demonstrated that GFAP levels were consistently altered in the cerebrospinal fluid (CSF) of symptomatic patients with AD, but studies of blood GFAP present relatively high variability.^17^

It is not yet well known how plasma GFAP levels change across the overall AD continuum and whether GFAP concentrations in CSF and blood reflect the same pathologic processes because reactive astrocytes assume multiple states—the so-called astrocyte heterogeneity. Thus, our main aim was to evaluate the levels of plasma GFAP throughout the AD continuum and compare them with the levels of CSF GFAP, with particular attention to preclinical AD. We hypothesized that plasma GFAP levels are already higher early in the preclinical stage and further elevated in symptomatic stages.

## Methods

### Study Population

This cross-sectional study, which included participants from 3 cohorts, collected data from July 29, 2014, to January 31, 2020. The Translational Biomarkers in Aging and Dementia (TRIAD) cohort (Montreal, Canada)^18^ comprised 300 individuals (177 women [59.0%]; mean [SD] age, 64.6 [17.6] years), including young CU adults, elderly CU adults, individuals with mild cognitive impairment (MCI), and patients with AD dementia. The ALFA+ cohort (Barcelona, Spain),^19^ which is a nested study of the ALFA (for Alzheimer’s and Families) study, included 384 middle-aged CU individuals (234 women [60.9%]; mean [SD] age, 61.1 [4.7] years) at elevated risk for AD. The BioCogBank Paris Lariboisière cohort (Paris, France)^20^ included 166 patients with cognitive disorders from the Center of Cognitive Neurology, Lariboisière Hospital, as well as 21 CU individuals. In addition to clinical classification (CU, MCI, and dementia), participants were categorized according to Aβ status (Aβ-positive [Aβ+] and Aβ-negative [Aβ–]), defined by results of Aβ positron emission tomography (PET) in TRIAD and the CSF Aβ42/40 ratio in ALFA+ and Paris, if not otherwise specified. ALFA+ participants were also classified using the AT (Aβ and tau pathology) classification.^21,22^ Participants with non–AD dementia (frontotemporal dementia [FTD] or dementia with Lewy bodies) from the TRIAD and Paris cohorts were included for supplementary analysis. All studies have been approved by their regional ethical committees (TRIAD: McGill University and Douglas Hospital Research Centre institutional review boards; ALFA+: Independent Ethics Committee “Parc de Salut Mar,” Barcelona; and Paris Cohort: Bichat Ethics Comittee), and all participants provided written informed consent. Additional details of the 3 cohorts are reported in the eMethods in Supplement 1.

### Fluid and Neuroimaging Biomarkers

Plasma and CSF samples from the 3 cohorts were independently analyzed at the Clinical Neurochemistry Laboratory, University of Gothenburg, Gothenburg, Sweden. Plasma and CSF GFAP levels were quantified for all cohorts on the Simoa HD-X (Quanterix) using the commercial single-plex assay (No. 102336). A comprehensive description of the fluid and neuroimaging biomarker measurements can be found in the eMethods in Supplement 1.

### Statistical Analysis

We used linear regression models to assess the association between plasma or CSF GFAP levels and the other biomarkers. Similar models were applied to evaluate group differences and associations with age and sex; the Tukey honestly significant difference test was used for post hoc pairwise comparisons. Fold changes and the effect size of the differences (estimated with Cohen *d* ) were calculated using Aβ– CU (CU–) individuals (TRIAD and Paris) and Aβ– and tau– (A–T–) individuals or Aβ− individuals (ALFA+) as reference groups. All analyses were adjusted for age and sex if not otherwise specified. The Spearman rank test was used for correlations using raw biomarker values. Receiver operating curve (ROC) analyses provided the area under the curve (AUC) for Aβ positivity or diagnostic groups. The “pROC” package in R, version 3.6.3 (R Group for Statistical Computing) was used to compare AUCs, and the false discovery rate was used to correct *P* values for multiple comparisons. Mediation analyses were performed with the R package “mediation.” All tests were 2-tailed, with a significance level of α = .05. All statistical analyses and figures were performed with R, version 3.6.3. Further details are provided in the eMethods in Supplement 1.

## Results

### Participants’ Characteristics and Correlations Between Biomarkers

Demographic and clinical data from the 3 studies are summarized in Table 1 and eTable 1 in Supplement 1. There was a positive association between age and both plasma and CSF GFAP levels in the 3 cohorts (TRIAD: plasma, β [SE] = 0.64 [0.13]; *P* < .001; CSF, β [SE] = 0.35 [0.15]; *P* = .02); ALFA+: plasma, β [SE] = 0.38 [0.048]; *P* < .001; CSF, β [SE] = 0.26 [0.049]; *P* < .001; and Paris: plasma, β [SE] = 0.26 [0.06]; *P* < .001; CSF, β [SE] = 0.32 [0.07]; *P* < .001), which can also be evidenced when comparing plasma or CSF GFAP mean levels between young CU participants and elderly CU– individuals (TRIAD: plasma, CU– mean [SD], 185.1 [93.5] pg/mL; young CU mean [SD], 95.1 [62.1] pg/mL; *P* = .001; CSF, CU– mean [SD], 12 506 [5148] pg/mL; young CU mean [SD], 4134 [1483] pg/mL; *P* < .001). Plasma GFAP levels were higher in CU women than in CU men (TRIAD: mean [SD], 161.0 [81.7] pg/mL in men vs 239.01 [123.84] pg/mL in women; *P* < .001; ALFA+: mean [SD], 128.9 [59.7] pg/mL in men vs 145.6 [63.1] pg/mL in women; *P* < .001) and were also higher specifically in CU– women compared with CU– men (TRIAD: mean [SD], 142.5 [63.2] pg/mL in men vs 209.1 [99.5] pg/mL in women; *P* < .001; ALFA+: mean [SD], 117.0 [43.9] pg/mL in men vs 125.1 [41.2] pg/mL in women; *P* = .01; and Paris cohort: mean [SD], 118.9 [34.6] pg/mL in men vs 179.34 [68.26] pg/mL in women; *P* = .03). The same sex differences were also observed when all participants were included (adjusting for age and diagnosis, TRIAD: mean [SD], 224.7 [153.2] pg/mL in men vs 248.1 [146.1] pg/mL in women; *P* = .002; Paris: mean [SD], 262.7 [138.4] pg/mL in men vs 326.7 [189.6] pg/mL in women; *P* < .001). *APOE *ε4 carriership (NCBI Gene ID: 348) was not associated with plasma or CSF GFAP levels in any of the cohorts when models accounted for Aβ status or clinical diagnosis.

**Table 1.** Demographic Characteristics and Biomarker Levels of the Study Cohorts by Clinical and Biomarker-Defined Groups^a^

Characteristic	TRIAD cohort (n = 300)							ALFA+ cohort (n = 384)			BioCogBank Paris Lariboisière cohort (n = 187)				
	Mean (SD)						*P* value	Mean (SD)		*P* value	Mean (SD)				*P* value
	Young CU (n = 35)	CU− (n = 114)	CU+ (n = 42)	MCI+ (n = 39)	AD dementia (n = 45)	Non-AD (n = 25)^b^		CU− (n = 249)^c^	CU+ (n = 135)^d^		CU− (n = 21)	MCI+ (n = 42)	AD dementia (n = 76)	Non-AD (n = 48)^e^	
Age, y	23.1 (1.8)	69.9 (9.4)	74.1 (7.7)	71.2 (7.7)	66.1 (9.7)	70.8 (11.0)	<.001	60.5 (4.5)	62.2 (4.9)	<.001	64.4 (9.5)	72.4 (7.9)	72.2 (8.4)	66.6 (9.7)	.001
Female, No. (%)	22 (62.9)	73 (64.0)	29 (69.0)	21 (53.8)	21 (46.7)	11 (44.0)	.12	153 (61.4)	81 (60.0)	.87	14 (66.7)	26 (61.9)	47 (61.8)	29 (60.4)	.97
Educational level, y	16.6 (1.5)	15.6 (3.9)	14.8 (3.2)	15.2 (3.2)	14.6 (3.6)	13.8 (3.9)	.02	13.6 (3.5)	13.3 (3.6)	.49	11.2 (1.6)	10.7 (1.8)	9.7 (2.0)	10.7 (1.9)	.004
*APOE* ε4 carriers, No. (%)	8 (22.9)	29 (26.9)	12 (28.6)	23 (62.2)	24 (55.8)	5 (22.7)	<.001	106 (42.6)	103 (76.3)	<.001	6 (28.6)	24 (57.1)	49 (64.5)	7 (14.6)	<.001
MMSE score	30 (0)	29 (1.0)	29 (1.0)	28 (2.0)	19 (6.0)	27 (2.0)	<.001	29.1 (0.9)	29.1 (1.0)	.93	27.4 (2.5)	23.5 (4.4)	19.3 (5.6)	24.6 (3.7)	<.001
Centiloids	−11.6 (6.6)	−3.12 (8.6)	52.5 (31.2)	91.1 (36.0)	91.8 (40.0)	1.10 (12.3)	<.001	−4.54 (6.6)	16.8 (21.1)	<.001	NA	NA	NA	NA	NA
**CSF biomarkers, pg/mL**															
Aβ42/40	0.091 (0.006)	0.087 (0.017)	0.055 (0.015)	0.043 (0.010)	0.045 (0.011)	0.082 (0.026)	<.001	0.087 (0.009)	0.051 (0.012)	<.001	0.095 (0.007)	0.044 (0.009)	0.042 (0.009)	0.089 (0.012)	<.001
p-tau181	22.6 (7.1)	36.2 (14.4)	59.3 (35.2)	89.4 (34.6)	99.9 (55.8)	59.7 (63.5)	<.001	13.9 (4.2)	18.4 (7.2)	<.001	32.8 (8.6)	93.0 (46.9)	115.4 (59.3)	37.7 (16.4)	<.001
t-tau	195.3 (48.1)	311.0 (126.8)	396.4 (197.0)	539.4 (210.1)	659.6 (331.7)	448.4 (398.6)	.001	174.8 (48.0)	222.6 (76.9)	<.001	243.1 (70.9)	587.6 (280.3)	732.6 (390.7)	305.6 (148.6)	<.001
NfL	184.6 (57.7)	1132.3 (1038.3)	862.5 (268.7)	1126.8 (257.7)	1646.2 (965.0)	1783.0 (1662.5)	.07	76.3 (23.6)	89.2 (27.5)	<.001	889.3 (352.1)	1532 (643.4)	1695 (673.0)	1456 (1214)	.03
GFAP	4134 (1483)	12 506 (5148)	15 669 (6771)	17 114 (5890)	16 314 (8513)	14 074 (7497)	.02	4090 (2018)	4859 (2333)	.01	2423 (2194)	4189 (3313)	4601 (3759)	2872 (2356)	.14
**Plasma biomarkers, pg/mL**															
NfL	6.5 (2.7)	22.1 (9.8)	27.9 (24.8)	25.7 (14.4)	33.6 (13.5)	28.6 (11.4)	<.001	9.8 (3.3)	11.6 (4.2)	<.001	13.1 (6.8)	24.2 (10.4)	24.4 (8.7)	21.2 (16.7)	.06
p-tau181	7.9 (3.6)	9.9 (4.4)	14.8 (11.0)	18.1 (8.1)	24.1 (9.6)	11.8 (12.3)	<.001	8.8 (3.2)	11.0 (4.6)	<.001	3.0 (1.8)	11.5 (6.2)	12.8 (3.6)	9.5 (6.7)	<.001
GFAP	95.1 (62.1)	185.1 (93.5)	285.0 (142.6)	332.5 (153.6)	388.1 (152.8)	188.9 (105.9)	<.001	121.9 (42.4)	169.9 (78.5)	<.001	161.2 (67.1)	368.6 (158.5)	376.4 (179.6)	185.0 (96.0)	<.001

Abbreviations: Aβ, amyloid-β; AD, Alzheimer disease; ALFA, Alzheimer’s and Families; CSF, cerebrospinal fluid; CU−, Aβ-negative cognitively unimpaired; CU+, Aβ-positive cognitively unimpaired; GFAP, glial fibrillary acidic protein, MCI+, Aβ-positive mild cognitive impairment; MMSE, Mini-Mental State Examination; NA, not available; NfL, neurofilament light chain; p-tau181, tau phosphorylated at threonine 181; t-tau, total tau; TRIAD, Translational Biomarkers in Aging and Dementia.

^a^ Within each cohort, we used *t* test or 1-way analysis of variance to compare age, educational level, and MMSE between groups and Pearson χ^2^ to compare sex and *APOE* ε4 frequencies between groups. Centiloids and fluid biomarker levels were compared with a 1-way analysis of covariance adjusted by age and sex and followed by false discovery rate multiple comparison correction. Aβ status for group definition was based on positron emission tomography visual result in the TRIAD cohort and on CSF Aβ42/40 for the ALFA+ and BioCogBank Paris Lariboisière cohorts.

^b^ Among the non-AD group, there were 21 individuals with MCI with a negative Aβ positron emission tomography visual result and 4 participants with a clinical diagnosis of AD dementia syndrome with a negative Aβ positron emission tomography visual result.

^c^ A total of 68 of 248 participants (27.4%) had subjective cognitive decline.

^d^ A total of 39 of 135 participants (28.9%) had subjective cognitive decline.

^e^ In the non-AD group all participants had MCI with normal CSF Aβ42/40 levels.

There was a positive correlation between plasma and CSF GFAP levels in the 3 cohorts (eFigure 1 in Supplement 1). Spearman rank correlations between plasma and CSF GFAP levels and other biomarkers are presented in eFigure 2 in Supplement 1.

### Plasma GFAP Levels Throughout the AD Continuum

**Figure 1.**
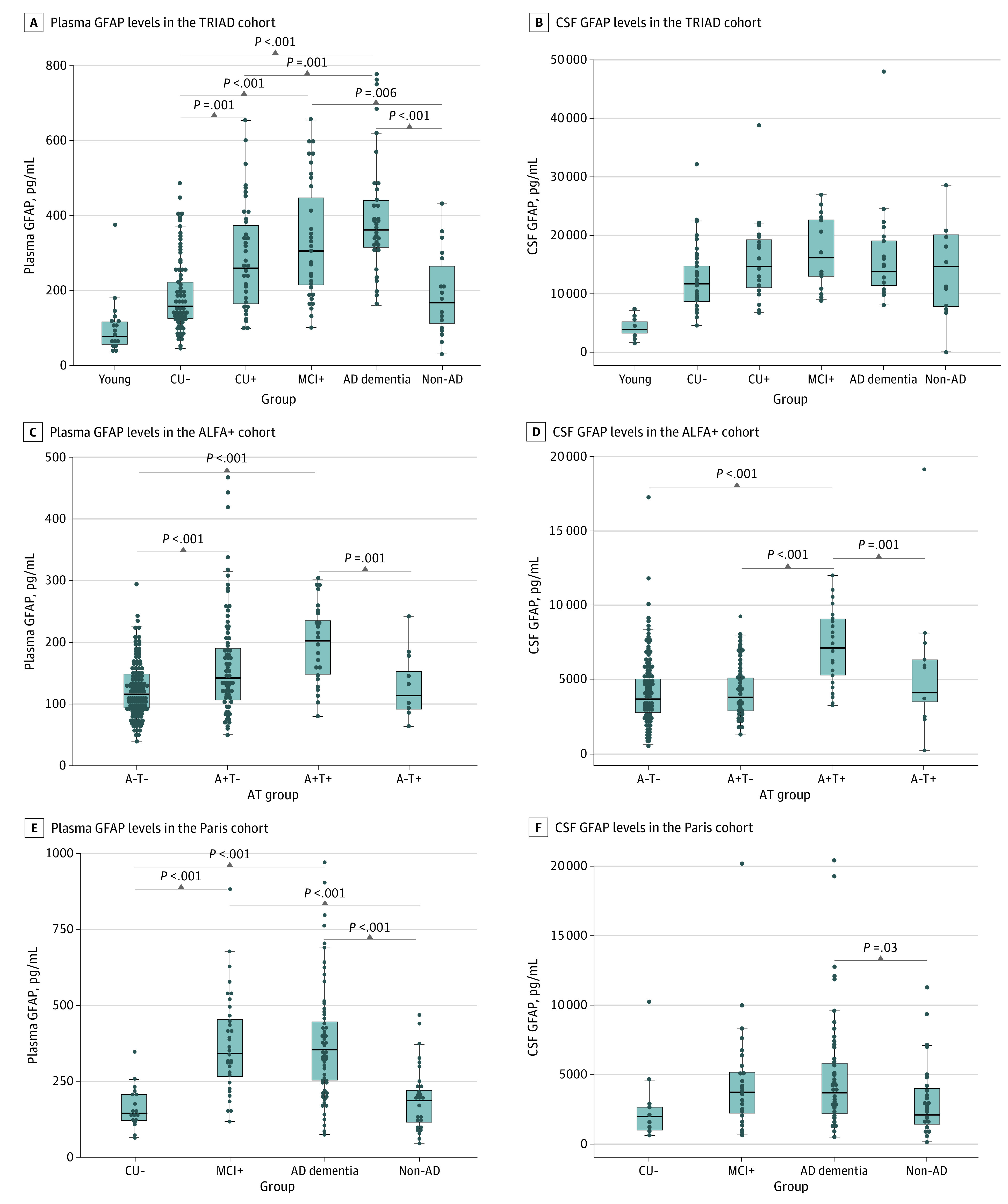
Plasma and Cerebrospinal Fluid (CSF) Glial Fibrillary Acidic Protein (GFAP) Group Comparisons Box plots depict median (horizontal bar), IQR (hinges), and 1.5 × IQR (whiskers). Group comparisons were computed with a 1-way analysis of covariance adjusting for age and sex. The Tukey honestly significant difference test was used for post hoc pairwise comparisons in all cohorts. Fold changes are depicted for the Alzheimer disease (AD) continuum groups and were calculated using amyloid-β (Aβ)–negative cognitively unimpaired (CU−) individuals (Translational Biomarkers in Aging and Dementia [TRIAD] and BioCogBank Paris Lariboisière [Paris] cohorts) or Aβ-negative and tau-negative (A–T–) individuals (Alzheimer’s and Families [ALFA+] cohort) as the reference group. Aβ status was defined by Aβ positron emission tomography in the TRIAD cohort and CSF Aβ42/40 ratio in the ALFA+ and Paris cohorts. The non-AD group included 21 individuals with Aβ-negative mild cognitive impairment (MCI), 4 individuals with Aβ-negative AD dementia syndrome in the TRIAD cohort, and 48 individuals with MCI− in the Paris cohort. A+T– indicates Aβ-positive and tau-negative; A+T+, Aβ-positive and tau-positive; A–T+, Aβ-negative and tau-positive; CU+, Aβ-positive cognitively unimpaired; MCI+, Aβ-positive MCI.

In the TRIAD cohort, levels of plasma and CSF GFAP were higher across the AD continuum, namely, in Aβ+ CU (CU+) individuals (ie, preclinical AD), individuals with Aβ+ MCI (MCI+; ie, MCI due to AD), and individuals with AD dementia (Figure 1A). Compared with the CU– group, plasma GFAP levels were higher in the CU+ group (54% increase; *P* = .001; *d* = 0.66), in the MCI+ group (79% increase; *P* < .001; *d* = 1.35), and in the AD dementia group (107% increase; *P* < .001; *d* = 2.10). Patients with FTD had plasma GFAP levels as low as CU– individuals (eFigure 3A in Supplement 1). Levels of CSF GFAP were also higher in the AD continuum groups compared with CU– individuals (Figure 1B), but the group differences were not significant after correction for multiple comparisons. The magnitude of the CSF GFAP changes was not as large as that of the plasma GFAP changes (the CSF GFAP level increases with CU– individuals as the reference group: CU+ individuals, 24% increase; *P* = .24; *d* = 0.56; individuals with MCI+, 35% increase; *P* = .06; *d* = 0.82; and individuals with AD dementia, 30% increase; *P* = .03; *d* = 0.86). Similar to plasma GFAP levels, patients with FTD had lower CSF GFAP levels than patients on the AD continuum (eFigure 3B in Supplement 1).

In ALFA+, we used the biomarker-based AT classification^21,22^ to study 2 stages in preclinical AD: Aβ+ but tau– (A+T–) and Aβ+ and tau+ (A+T+) and compared it with the A–T– stage. Plasma GFAP levels were significantly higher in the A+T– group compared with the A–T– group (32% increase; *P* < .001; *d* = 0.55) (Figure 1C), whereas CSF GFAP levels were not (1% increase; *P* = .99; *d* = 0.01; Figure 1D). Both plasma and CSF GFAP were significantly higher in the A+T+ group compared with the A–T– group (plasma: 60% increase; *P* < .001; *d* = 1.09; CSF: 77% increase; *P* < .001; *d* = 1.18). Participants in the Aβ– and tau+ (A–T+ group) did not have higher plasma or CSF GFAP levels compared with the A–T– group. To further test whether plasma and CSF GFAP levels were increased in the earliest stage of the preclinical AD continuum, we analyzed a group of individuals with a low burden of Aβ pathology, namely, a positive CSF Aβ42/40 ratio but Aβ PET centiloids lower than 30^23^ (eMethods in Supplement 1). We observed that plasma GFAP levels were significantly higher in this group compared with Aβ– participants (28% increase; *P* < .001; *d* = 0.57; eFigure 4A in Supplement 1) while CSF GFAP levels were not (8% increase; *P* = .37; *d* = 0.16; eFigure 4B in Supplement 1).

In the Paris cohort, plasma and CSF GFAP levels followed similar patterns to those described for TRIAD. Plasma GFAP levels were higher in individuals with MCI+ (128% increase; *P* < .001; *d* = 1.40) and in those with AD dementia (133% increase; *P* < .001; *d* = 1.37) compared with the CU– group, and no difference was found between the CU– group and non-AD group (Figure 1E). Levels of CSF GFAP were higher in individuals with MCI+ (72% increase; *d* = 0.44) and AD dementia (89% increase; *d* = 0.64) compared with CU– individuals, but differences were not statistically significant after correction for multiple comparisons (Figure 1F). Similar to TRIAD, patients with FTD and dementia with Lewy bodies had plasma and CSF GFAP levels comparable to CU– individuals (eFigure 3C and 3D in Supplement 1).

### Association of Plasma GFAP Levels With Aβ Pathology and Discrimination of Aβ Status

**Figure 2.**
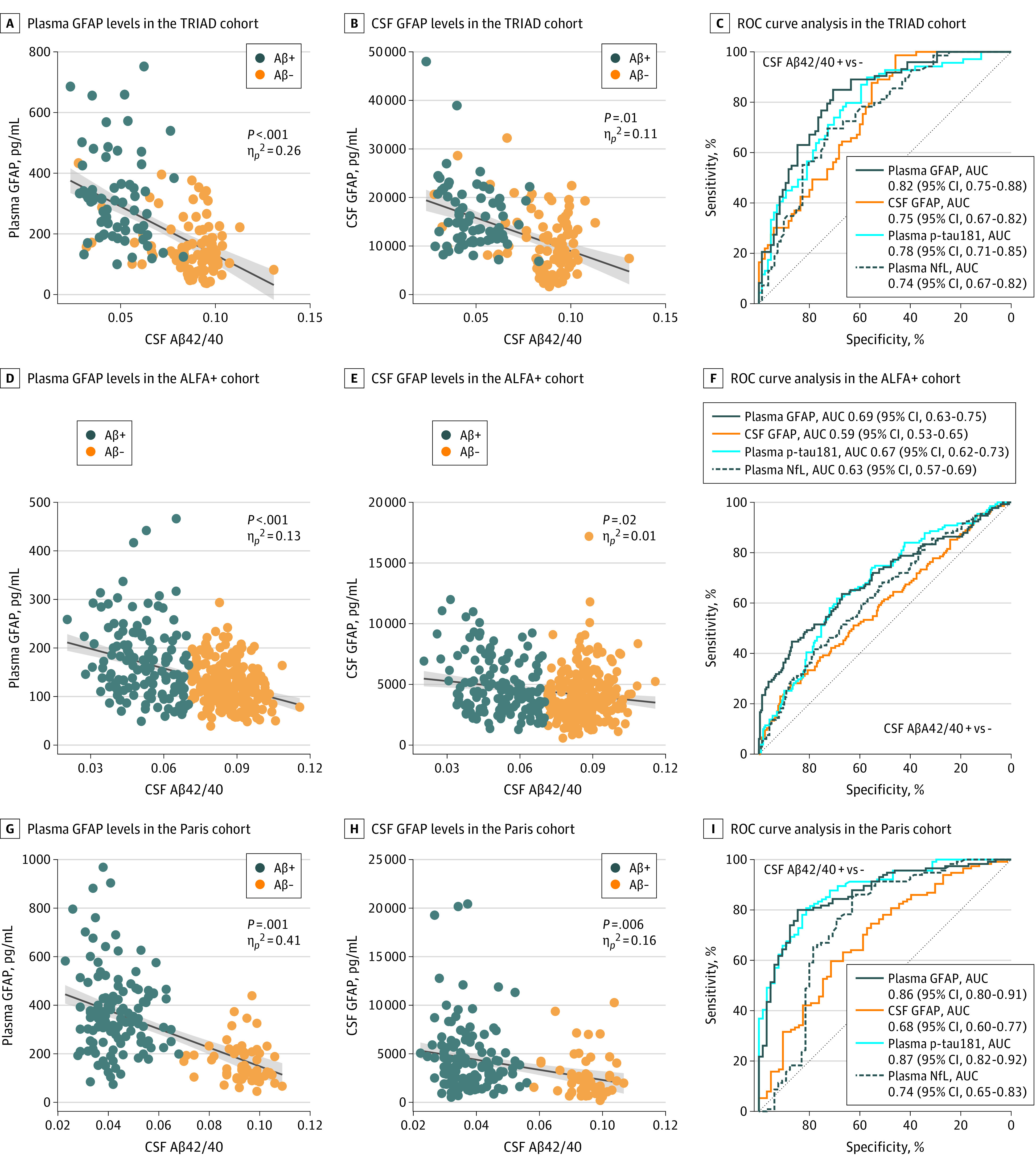
Associations of Plasma and Cerebrospinal Fluid Glial Fibrillary Acidic Protein Levels With Aβ Pathology and Discriminative Accuracy Individuals are color coded by amyloid-β (Aβ) status (as defined by Aβ positron emission tomography in the Translational Biomarkers in Aging and Dementia [TRIAD] cohort and cerebrospinal fluid (CSF) Aβ42/40 ratio in the Alzheimer’s and Families [ALFA+] and BioCogBank Paris Lariboisière [Paris] cohorts). Solid lines indicate the regression line and 95% CIs. *P* values were computed with linear models adjusted by age, sex, and clinical diagnosis (the latter only for the TRIAD and Paris cohorts). Sizes of the associations between variables are shown by the partial η^2^ (η*_p_*^2^). For comparative purposes, we also included plasma tau phosphorylated at threonine 181 (p-tau181) and plasma neurofilament light chain (NfL) in these analyses. AUC indicates area under the curve; GFAP, glial fibrillary acidic protein; ROC, receiver operating characteristic.

We evaluated the association of plasma and CSF GFAP levels with Aβ pathology as measured with CSF Aβ42/40 or Aβ PET. Because our aim was to study the AD continuum, for all subsequent analyses, we included only CU individuals, those with MCI, and those with AD dementia (for TRIAD and Paris cohorts). In the ALFA+ cohort, we excluded individuals with an A–T+ (non-AD pathologic change) biomarker profile. In TRIAD, both plasma and CSF GFAP levels were negatively associated with CSF Aβ42/40 (plasma GFAP, *P* < .001; η*_p_*^2^ = 0.26; CSF GFAP, *P* = .01; η*_p_*^2^ = 0.11; Figure 2A and B) and positively associated with Aβ PET (plasma GFAP, *P* < .001; η*_p_*^2^ = 0.32; CSF GFAP, *P* < .001; η*_p_*^2^ = 0.10; eFigure 5A and 5B in Supplement 1). The sizes of the associations of Aβ pathology (either CSF Aβ42/40 or Aβ PET) with plasma GFAP levels were larger than those with CSF GFAP levels. We performed the same analyses within the CU individuals, and plasma GFAP levels were significantly associated with both Aβ biomarkers (CSF Aβ42/40: *P* = .008; η*_p_*^2^ = .07; Aβ PET: *P* < .001; η*_p_*^2^ = .06). In contrast, CSF GFAP levels were not significantly associated with CSF Aβ42/40 (*P* = .18) or Aβ PET (*P* = .07) within the CU individuals.

In ALFA+, plasma GFAP levels were positively associated with Aβ pathology as shown by a significant negative association with CSF Aβ42/40 in the whole sample (*P* < .001; η*_p_*^2^ = 0.13) but also in the CU– group (*P* = .002; η*_p_*^2^ = 0.04) and CU+ group (*P* = .03; η*_p_*^2^ = 0.04) (Figure 2D). Levels of CSF GFAP also showed a negative association with CSF Aβ42/40 in the whole sample (*P* = .02; η*_p_*^2^ = 0.01; Figure 2E) and in the CU+ group (*P* = .005; η*_p_*^2^ = 0.06). Conversely, a positive association between CSF GFAP levels and CSF Aβ42/40 was observed in CU– participants (*P* = .02; η*_p_*^2^ = 0.02). Both plasma and CSF GFAP levels were associated with Aβ deposition as quantified by Aβ PET (eFigure 5C and D in Supplement 1) in the whole sample (plasma GFAP, *P* < .001; η*_p_*^2^ = 0.10; CSF GFAP, *P* = .001; η*_p_*^2^ = 0.04).

The same analysis was repeated in the Paris cohort, and the size of the association of CSF Aβ42/40 with plasma GFAP levels (plasma, *P* < .001; η*_p_*^2^ = 0.41) was greater than that with CSF GFAP levels (CSF, *P* = .006; η*_p_*^2^ = 0.16; Figure 2G and H).

We next investigated how plasma and CSF GFAP levels discriminate Aβ status using ROC analysis (Table 2 and Figure 2). Aβ statuses were defined by CSF Aβ42/40, Aβ PET visual read, or the Aβ PET centiloids cutoffs used in each cohort (Table 2). In the entire TRIAD cohort, plasma GFAP as a biomarker accurately discriminated Aβ+ from Aβ− individuals, with an AUC ranging from 0.82 to 0.85. In contrast, CSF GFAP as a biomarker had an AUC of 0.75. In CU individuals, plasma GFAP as a biomarker distinguished Aβ status with an AUC of 0.75 to 0.79, whereas CSF GFAP as a biomarker had AUCs of 0.74 to 0.76. In ALFA+, plasma GFAP as a biomarker discriminated with an AUC of 0.69 to 0.82, while for CSF GFAP as a biomarker, AUCs were 0.59 to 0.76. In the Paris cohort, plasma GFAP as a biomarker accurately differentiated CSF Aβ42/40 status with an AUC of 0.86, while CSF GFAP as a biomarker had an AUC of 0.68. In addition, ROCs were performed contrasting CU– individuals with those with MCI+, individuals with Aβ– MCI (MCI–) with those with MCI+, and CU– individuals with those with AD (eTable 2 in Supplement 1). For comparison purposes, we also performed ROC analyses with plasma tau phosphorylated at threonine 181 (p-tau181) and neurofilament light chain (NfL), and none of them performed better than plasma GFAP.

**Table 2.** ROC Curve Analyses to Discriminate Aβ-Positive From Aβ-Negative Individuals

Biomarker	Aβ+ vs Aβ−, AUC (95% CI)^a^						
	CSF Aβ42/40			Aβ PET			
	TRIAD cohort	ALFA+ cohort	BioCogBank Paris Lariboisière cohort	Visual result		Centiloid cutoff	
				TRIAD cohort	ALFA+ cohort	TRIAD cohort	ALFA+ cohort
GFAP							
Plasma	0.82 (0.75-0.88)	0.69 (0.63-0.75)	0.86 (0.80-0.91)	0.85 (0.79-0.91)	0.75 (0.67-0.84)	0.83 (0.77-0.89)	0.82 (0.72-0.92)
CSF	0.75 (0.67-0.82)^b^	0.59 (0.53-0.65)^c^	0.68 (0.60-0.77)^c^	0.75 (0.69-0.82)^c^	0.68 (0.59-0.77)	0.75 (0.68-0.84)^d^	0.76 (0.64-0.87)
Other plasma biomarkers							
p-tau181	0.78 (0.71-0.85)	0.67 (0.62-0.73)^e^	0.87 (0.82-0.92)^e^	0.77 (0.70-0.85)	0.67 (0.58-0.76)	0.79 (0.71-0.86)	0.76 (0.67-0.86)
NfL	0.74 (0.67-0.82)	0.63 (0.57-0.69)	0.74 (0.65-0.83)^c^	0.67 (0.59-0.76)^c^	0.66 (0.58-0.75)	0.68 (0.59-0.76)^c^	0.73 (0.63-0.83)

Abbreviations: Aβ, amyloid-β; ALFA, Alzheimer’s and Families; AUC, area under the curve; CSF, cerebrospinal fluid; GFAP, glial fibrillary acidic protein; NfL, neurofilament light chain; p-tau181, tau phosphorylated at threonine 181; PET, positron emission tomography; ROC, receiver operating characteristic; TRIAD, Translational Biomarkers in Aging and Dementia.

^a^ ROC curve analyses to test whether plasma GFAP discriminates between Aβ-positive (Aβ+) and Aβ-negative (Aβ−) individuals, as defined by the CSF Aβ42/40 ratio, Aβ PET visual result, or Aβ PET using a cutoff of 24 (TRIAD) or 30 (ALFA) centiloids. We also included CSF GFAP, plasma p-tau181, and plasma NfL for comparison. AUC differences were tested using the DeLong test followed by false discovery rate multiple comparison correction.

^b^ *P* = .06 vs plasma GFAP (before correction for multiple comparisons).

^c^ *P* < .05 vs plasma GFAP.

^d^ *P* = .03 vs plasma GFAP (before correction for multiple comparisons).

^e^ *P* < .05 vs CSF GFAP.

We also performed analyses comparing different combinations of plasma biomarkers (eTable 3 in Supplement 1). We found that adding plasma GFAP to any of the other plasma biomarkers (either p-tau181 or NfL) was associated with improved accuracy to discriminate Aβ status (as measured by CSF Aβ42/40) in the 3 cohorts.

### Association of Plasma GFAP Levels With Tau Pathology Among Individuals With Concomitant Aβ Pathology

**Figure 3.**
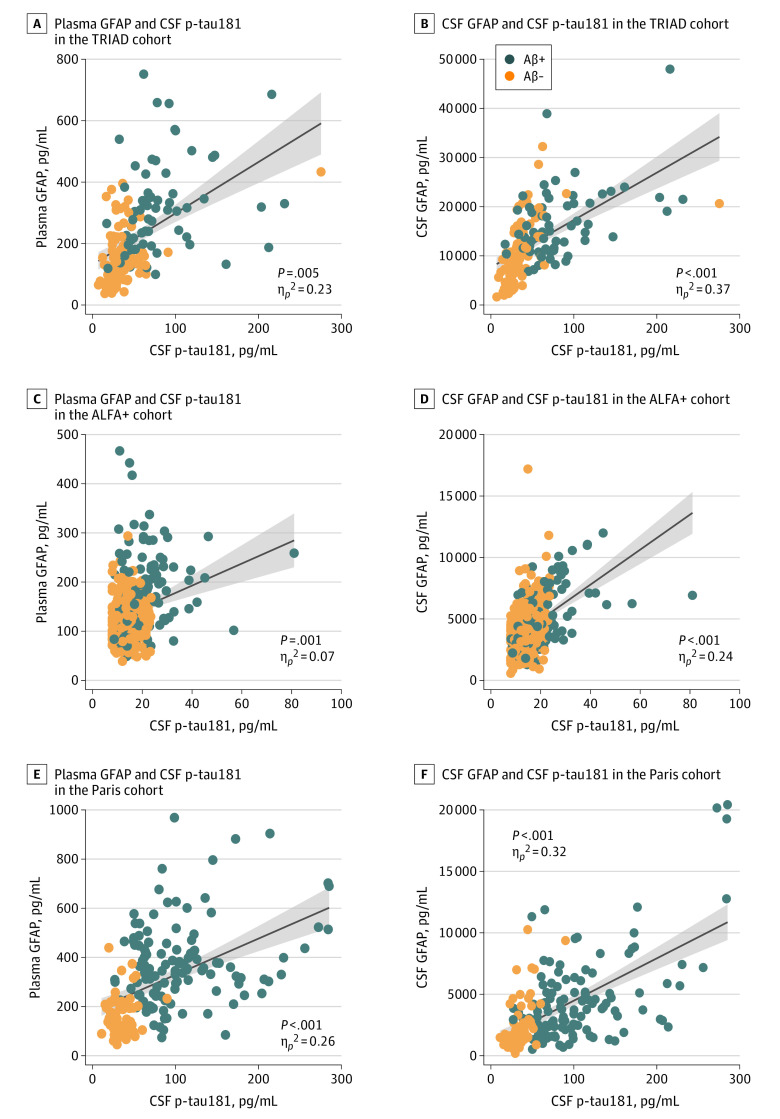
Association of Plasma and Cerebrospinal Fluid (CSF) Glial Fibrillary Acidic Protein (GFAP) Levels With Tau Phosphorylated at Threonine 181 (p-tau181) A, Association of plasma GFAP with CSF p-tau181 in the Translational Biomarkers in Aging and Dementia (TRIAD) cohort. B, Association of CSF GFAP with CSF p-tau181 in the TRIAD cohort. C, Association of plasma GFAP with CSF p-tau181 in the Alzheimer’s and Families (ALFA+) cohort. D, Association of CSF GFAP with CSF p-tau181 in the ALFA+ cohort. E, Association of plasma GFAP with CSF p-tau181 in the BioCogBank Paris Lariboisière (Paris) cohort. F, Association of CSF GFAP with CSF p-tau181 in the Paris cohort. Individuals are colored by amyloid-β (Aβ) status (as defined by Aβ positron emission tomography in the TRIAD cohort or CSF Aβ42/40 in the ALFA+ and Paris cohorts). The solid lines indicate the regression line and the 95% CIs. *P* values were computed with linear models adjusted by age, sex, and clinical diagnosis (the latter only for the TRIAD and Paris cohorts). The sizes of the associations between variables are shown by the partial η^2^ (η*_p_*^2^).

We evaluated the associations between GFAP levels and tau biomarkers (CSF p-tau181 and tau PET). In TRIAD, higher plasma and CSF GFAP levels were associated with increased tau PET burden (plasma GFAP, *P* < .001; η*_p_*^2^ = 0.29; CSF GFAP, *P* = .005; η*_p_*^2^ = 0.08; eFigure 6A and B in Supplement 1). Both plasma and CSF GFAP levels were significantly associated with CSF p-tau181 levels in the 3 cohorts (Figure 3A-F).

We conducted a mediation analysis to assess whether the associations between GFAP levels and tau biomarkers were mediated by Aβ status. Results in TRIAD indicated that the association of plasma GFAP levels with tau was mediated by Aβ (eFigure 7A in Supplement 1), with a significant indirect association corresponding to 60% of the total association of tau with plasma GFAP levels. These findings were replicated using PET biomarkers (eFigure 7A in Supplement 1). A similar analysis was performed with CSF GFAP levels as the response variable, and tau had both a direct and an indirect association with CSF GFAP levels.

Results were consistent across cohorts (eFigure 7B and C in Supplement 1). In the ALFA+ and Paris cohorts, the association of CSF p-tau181 with plasma GFAP levels was mediated by CSF Aβ42/40, with a significant indirect association corresponding to 62% and 63% of the total association of CSF p-tau181 with plasma GFAP levels, respectively. Conversely, CSF p-tau181 did not show a significant indirect association with CSF GFAP levels, suggesting Aβ-independent effects.

### Association of CSF and Plasma GFAP Levels With Neuroinflammation

Finally, we explored how plasma and CSF GFAP levels are associated with other glial biomarkers. In TRIAD, levels of CSF GFAP, but not plasma GFAP, showed a positive association with CSF soluble triggering receptor expressed on myeloid cells 2 (sTREM2) and Chitinase-3-like protein 1 (YKL40) (TRIAD: plasma GFAP association with sTREM2, β [SE] = 0.11 [0.08]; *P* = .17; YKL40, β [SE] = 0.02 [0.06]; *P* = .67; CSF GFAP association with sTREM2, β [SE] = 0.25 [0.09]; *P* < .001; YKL40, β [SE] = 0.32 [0.07]; *P* < .001) (eFigure 8A and B in Supplement 1). Similar results were observed in the ALFA+ and Paris cohorts (ALFA+: plasma GFAP association with sTREM2, β [SE] = 0.083 [0.086]; *P* = .14; YKL40, β [SE] = 0.075 [0.051]; *P* = .14; CSF GFAP association with sTREM2, β [SE] = 0.41 [0.048]; *P* < .001; YKL40, β [SE] = 0.40 [0.045]; *P* < .001; and Paris: plasma GFAP association with YKL40, β [SE] = 0.06 [0.09]; *P* = .49; CSF GFAP association with YKL40, β [SE] = 0.52 [0.12]; *P* < .001) (eFigure 8C-E in Supplement 1).

## Discussion

In this study, which includes 3 thoroughly characterized cohorts, we showed that plasma GFAP levels were significantly higher among individuals with preclinical AD and reached their higher levels at symptomatic stages of AD. The effect sizes of the increases of plasma GFAP levels were always larger than those of CSF GFAP levels. Therefore, plasma GFAP levels appear to be a superior biomarker tracking Aβ pathology than its CSF counterpart. This finding is particularly evident for individuals with preclinical AD; plasma GFAP levels were significantly higher in CU+ individuals and significantly discriminated them from CU– individuals, whereas CSF did not.

Previous studies showed that plasma and serum GFAP levels are higher in those with symptomatic AD,^9,24,25,26,27^ results that are in line with those reported for CSF GFAP levels.^24,28,29,30,31^ However, less is known about plasma GFAP levels among individuals along the whole AD continuum and, particularly, in those with preclinical AD. A recent study demonstrated that plasma GFAP levels were higher in a group of 33 CU+ individuals compared with 63 CU– individuals (AUC = 0.795).^16^ Preceding studies showed that plasma GFAP levels were associated with both clinical diagnosis and Aβ status.^25^ Another study revealed a quadratic (inverted U-shape) association between plasma GFAP levels and Aβ deposition.^26^ To our knowledge, no other studies investigated the whole AD continuum or included participants with preclinical AD, and no other studies compared plasma and CSF compartments in the same individuals.

We also analyzed the association of plasma GFAP levels with Aβ pathology (either CSF Aβ42/40 ratio or Aβ PET), and we found a positive association between plasma GFAP levels and Aβ pathology in all cohorts and high rates of accuracy to discriminate Aβ+ from Aβ– individuals (AUC = 0.82-0.86). It was also apparent when assessing the whole AD continuum that plasma GFAP levels were higher in individuals with a more advanced clinical diagnosis (CU+ less than MCI+, which was less than AD dementia). In contrast, CSF GFAP levels showed no significant difference across the AD continuum groups. Consistent with this finding, we observed a significant association between plasma GFAP levels and tau PET findings.

We included many individuals with preclinical AD: 42 in TRIAD and 135 in ALFA+. Plasma GFAP discriminated CU+ individuals from CU– individuals with an AUC of 0.75 to 0.79 in TRIAD, similar to the AUC of 0.795 previously described.^16^ Furthermore, in ALFA+, we studied the earliest phase of preclinical AD. We assessed 104 individuals who were A+T– (ie, had Aβ pathology but not yet tau pathology) and 89 individuals with a low Aβ burden (ie, they had decreased CSF Aβ42/40 but not yet a positive Aβ PET result). Both groups had significantly higher plasma GFAP levels but not CSF GFAP levels, reinforcing the idea that plasma GFAP may be an early biomarker of AD pathologic changes. Levels of CSF GFAP only become significantly higher in the A+T+ group when there is biomarker evidence of both Aβ and tau pathology. Data from cellular models indicate that astrocytes react to early preplaque-insoluble Aβ oligomeric species.^32^ Our results can be contextualized with findings using other fluid or imaging biomarkers of reactive astrogliosis. Studies using the PET tracer ^11^C-deuterium-L-deprenyl ([^11^C]DED), which binds to monoamine oxidase-B, mainly expressed in reactive astrocytes, support fluctuations during the AD continuum in reactive astrocyte states. More specifically, [^11^C]DED binding in the frontal and parietal cortices is significantly increased in those with prodromal AD compared with CU individuals.^33^ Early increases in [^11^C]DED binding have also been found in autosomal carriers of a dominant AD variation almost 30 years before the emergence of symptoms.^34^ In a transgenic mouse model that overexpresses the human *APPswe* variation, increased [^11^C]DED binding precedes detectable Aβ pathology.^35^ Moreover, CSF YKL40, a biomarker of a subset of reactive astrocytes, is also elevated in those with preclinical AD.^36,37^ Recently, a model of reactive astrogliosis in the AD continuum^15^ has been proposed that would encompass early reactive astrocytes in the preclinical stage (supported by in vivo evidence of higher monoamine oxidase-B expression), followed by more widespread reactivity (supported by increases in CSF YKL40, GFAP, and S100b) and, finally, the end-stage reactive astrocytes, in which their physiological function may be lost. Our findings situate plasma GFAP levels as a marker of early reactive astrocytes.

Our results point to plasma GFAP as a possible biomarker specific for Aβ pathology. First, plasma GFAP levels were not higher among individuals with non-AD neurodegenerative diseases in the TRIAD and Paris cohorts. Plasma GFAP levels were normal in those with FTD despite gliosis being a characteristic of FTD.^38,39^ Second, in ALFA+, the A–T+ group did not have high plasma GFAP levels; this finding may suggest that plasma GFAP levels specifically reflect Aβ pathology in preclinical stages, but a direct comparison with the preclinical stage of other neurodegenerative diseases should be performed. Third, the association between plasma GFAP levels and tau pathology was mediated by Aβ pathology. These results are consistent with the increased expression of GFAP surrounding Aβ plaques.^40,41,42,43^ Although CSF GFAP levels were associated with other glial biomarkers (YKL40 and sTREM2), plasma GFAP levels were not. It is possible that CSF GFAP better reflects reactive astrocytes in response to neuroinflammatory changes, such as microglial activation, while plasma GFAP is more closely associated with reactive astrogliosis because of Aβ burden. High levels of blood GFAP can be found in individuals with other neurodegenerative diseases,^24,44,45^ but this finding occurs at the symptomatic, and thus advanced, stages of the disease. The increase in blood GFAP levels after acute brain conditions, such as subarachnoid hemorrhage and traumatic and hypoxic brain injury, has been extensively documented,^46,47,48,49,50^ but this increase may come through other mechanisms, such as a trauma-induced temporary opening of the blood-brain barrier. Based on these findings, it would seem that GFAP responds to acute neuronal injury; however, in a chronic neurodegenerative disease, and unlike NfL, plasma GFAP may principally (but not exclusively) reflect Aβ pathology.

A unique feature of our study is that we measured both plasma and CSF GFAP levels in the same participants. This feature allowed us to draw one of the main conclusions of this study, namely, that differences in plasma GFAP levels are larger than those of CSF GFAP levels between the groups, and the effect sizes of the associations between plasma GFAP levels and biomarkers of Aβ are greater than those of CSF GFAP levels. Moreover, the AUCs to discriminate Aβ status are higher for plasma GFAP than CSF GFAP, especially when Aβ pathology is defined by CSF Aβ42/40, suggesting an early increase of plasma GFAP levels. This result is surprising because neurologically associated blood biomarkers have usually been considered a proxy of the CSF biomarkers. A possible explanation of why plasma GFAP outperforms CSF GFAP would be the different clearance mechanisms into the biofluids. Astrocytes are part of the neurovascular unit and the blood-brain barrier, which is altered in individuals with AD.^51^ Astrocytic end-feet cover brain capillaries, which may be a direct route for the release of GFAP from reactive astrocytes to the bloodstream.^52^ It could be speculated that blood-brain barrier dysfunction facilitates the release of GFAP into the bloodstream; this may also explain the elevations of plasma GFAP in individuals with acute neurologic injuries. Astrocytes are also part of the glymphatic system, which is a highly organized system that clears the brain of insoluble proteins and metabolites by draining them into the venous system.^53^ GFAP may also reach the bloodstream via the meningeal lymphatic system.^54^ Finally, preanalytical and analytical factors that need to be further studied may also account for these differences. A previous study described that plasma GFAP is very stable to freeze-thaw cycles,^55^ whereas CSF GFAP is far more sensitive over time.^56^ The fact that plasma GFAP has a wider range of values than CSF GFAP may also be associated with the higher accuracy of the former.

It remains unanswered which plasma biomarker (GFAP, Aβ42/40, or forms of p-tau) is more accurately associated with Aβ pathology in particular in the preclinical stage. A head-to-head comparison of these biomarkers in several independent cohorts is needed. However, GFAP is an additional tool that has shown consistent results across multiple cohorts and is easily detectable using commercially available immunoassays. Moreover, we show that adding plasma GFAP to models with other plasma biomarkers (p-tau181 and/or NfL) improves their accuracy. All of these biomarkers perform satisfactorily, but a combination of some will probably render the highest accuracy for Aβ pathology. This is particularly true in preclinical AD, when the individual increases of these biomarkers may be statistically significant, but the effect sizes of these increases are not large.

### Limitations

This study has some limitations. It is a cross-sectional study, and findings need to be confirmed with longitudinal data. The 3 cohorts have differences in the design and goals, and not all of them had the same data available. Also, the definitions of Aβ pathology differed between cohorts, which may limit comparability between them; however, the fact that the main results are validated in diverse studies confirms the robustness of our results. Finally, we did not include measurements of Aβ in blood.

## Conclusions

Altogether, these results suggest that high plasma GFAP levels are found early in the AD continuum and become greater during disease progression, in parallel with clinical syndrome severity and markers of tau pathology. Our findings have important implications in facilitating the detection of AD, particularly in its preclinical stage. This earlier detection may accelerate primary and secondary prevention trials and the design of interventional studies at early stages of AD. Plasma GFAP, alone or in combination with other biomarkers, could be used to screen for Aβ+ individuals at any stage across the AD continuum.

## Notes

Supplement 1. eTable 1. Demographic Characteristics and Biomarker Levels of the Study Cohorts by Clinical and Biomarker-Defined Groups Including Other non-AD Dementia groups (TRIAD and Paris Cohorts) eTable 2. ROC Analysis to Discriminate Diagnostic Groups in the Paris Cohort eTable 3. ROC Analyses of the Plasma Biomarker Combinations to Discriminate Aβ-Positive From Aβ-Negative Individuals eFigure 1. Correlations Between Plasma and CSF GFAP eFigure 2. Biomarker Correlations eFigure 3. Plasma and CSF GFAP Group Comparisons (Including Other Dementias) eFigure 4. Plasma and CSF GFAP Group Comparisons in Early Amyloid Accumulators eFigure 5. Plasma and CSF GFAP Associations With Amyloid PET eFigure 6. Plasma and CSF GFAP Associations With Tau PET eFigure 7. Aβ as a Mediator of the Association Between p-Tau and Plasma GFAP eFigure 8. Plasma and CSF GFAP Associations With Neuroinflammation Biomarkers eMethods. eReferences.

Supplement 2. Members of the Translational Biomarkers in Aging and Dementia (TRIAD) study, ALFA study, and BioCogBank Paris Lariboisière cohort

## References

[1] Janelidze S, Mattsson N, Palmqvist S, . Plasma p-tau181 in Alzheimer’s disease: relationship to other biomarkers, differential diagnosis, neuropathology and longitudinal progression to Alzheimer’s dementia. Nat Med. 2020;26(3):379-386. doi:10.1038/s41591-020-0755-1 32123385

[2] Karikari TK, Pascoal TA, Ashton NJ, . Blood phosphorylated tau 181 as a biomarker for Alzheimer’s disease: a diagnostic performance and prediction modelling study using data from four prospective cohorts. Lancet Neurol. 2020;19(5):422-433. doi:10.1016/S1474-4422(20)30071-5 32333900

[3] Thijssen EH, La Joie R, Wolf A, ; Advancing Research and Treatment for Frontotemporal Lobar Degeneration (ARTFL) investigators. Diagnostic value of plasma phosphorylated tau181 in Alzheimer’s disease and frontotemporal lobar degeneration. Nat Med. 2020;26(3):387-397. doi:10.1038/s41591-020-0762-2 32123386 PMC7101073

[4] Lantero Rodriguez J, Karikari TK, Suárez-Calvet M, . Plasma p-tau181 accurately predicts Alzheimer’s disease pathology at least 8 years prior to post-mortem and improves the clinical characterisation of cognitive decline. Acta Neuropathol. 2020;140(3):267-278. doi:10.1007/s00401-020-02195-x 32720099 PMC7423866

[5] Ashton NJ, Pascoal TA, Karikari TK, . Plasma p-tau231: a new biomarker for incipient Alzheimer’s disease pathology. Acta Neuropathol. 2021;141(5):709-724. doi:10.1007/s00401-021-02275-6 33585983 PMC8043944

[6] Palmqvist S, Janelidze S, Quiroz YT, . Discriminative accuracy of plasma phospho-tau217 for Alzheimer disease vs other neurodegenerative disorders. JAMA. 2020;324(8):772-781. doi:10.1001/jama.2020.12134 32722745 PMC7388060

[7] Janelidze S, Stomrud E, Palmqvist S, . Plasma β-amyloid in Alzheimer’s disease and vascular disease. Sci Rep. 2016;6(1):26801. doi:10.1038/srep26801 27241045 PMC4886210

[8] Verberk IMW, Slot RE, Verfaillie SCJ, . Plasma amyloid as prescreener for the earliest Alzheimer pathological changes. Ann Neurol. 2018;84(5):648-658. doi:10.1002/ana.25334 30196548 PMC6282982

[9] Simrén J, Leuzy A, Karikari TK, ; AddNeuroMed consortium. The diagnostic and prognostic capabilities of plasma biomarkers in Alzheimer’s disease. Alzheimers Dement. 2021;17(7):1145-1156. doi:10.1002/alz.12283 33491853 PMC8359457

[10] Nakamura A, Kaneko N, Villemagne VL, . High performance plasma amyloid-β biomarkers for Alzheimer’s disease. Nature. 2018;554(7691):249-254. doi:10.1038/nature25456 29420472

[11] Schindler SE, Bollinger JG, Ovod V, . High-precision plasma β-amyloid 42/40 predicts current and future brain amyloidosis. Neurology. 2019;93(17):e1647-e1659. doi:10.1212/WNL.0000000000008081 31371569 PMC6946467

[12] Keshavan A, Pannee J, Karikari TK, . Population-based blood screening for preclinical Alzheimer’s disease in a British birth cohort at age 70. Brain. 2021;144(2):434-449. doi:10.1093/brain/awaa403 33479777 PMC7940173

[13] Escartin C, Galea E, Lakatos A, . Reactive astrocyte nomenclature, definitions, and future directions. Nat Neurosci. 2021;24(3):312-325. doi:10.1038/s41593-020-00783-4 33589835 PMC8007081

[14] Verkhratsky A, Olabarria M, Noristani HN, Yeh CY, Rodriguez JJ. Astrocytes in Alzheimer’s disease. Neurotherapeutics. 2010;7(4):399-412. doi:10.1016/j.nurt.2010.05.017 20880504 PMC5084302

[15] Carter SF, Herholz K, Rosa-Neto P, Pellerin L, Nordberg A, Zimmer ER. Astrocyte biomarkers in Alzheimer’s disease. Trends Mol Med. 2019;25(2):77-95. doi:10.1016/j.molmed.2018.11.006 30611668

[16] Chatterjee P, Pedrini S, Stoops E, . Plasma glial fibrillary acidic protein is elevated in cognitively normal older adults at risk of Alzheimer’s disease. Transl Psychiatry. 2021;11(1):27. doi:10.1038/s41398-020-01137-1 33431793 PMC7801513

[17] Bellaver B, Ferrari-Souza JP, Uglione da Ros L, . Astrocyte biomarkers in Alzheimer disease: a systematic review and meta-analysis. Neurology. 2021;96(24):e2944-e2955. doi:10.1212/WNL.0000000000012109 33952650

[18] Pascoal TA, Shin M, Kang MS, . In vivo quantification of neurofibrillary tangles with [^18^F]MK-6240. Alzheimers Res Ther. 2018;10(1):74. doi:10.1186/s13195-018-0402-y 30064520 PMC6069775

[19] Molinuevo JL, Gramunt N, Gispert JD, . The ALFA Project: a research platform to identify early pathophysiological features of Alzheimer’s disease. Alzheimers Dement (N Y). 2016;2(2):82-92. doi:10.1016/j.trci.2016.02.003 29067295 PMC5644283

[20] Dumurgier J, Paquet C, Peoc’h K, . CSF Aβ_1-42_ levels and glucose metabolism in Alzheimer’s disease. J Alzheimers Dis. 2011;27(4):845-851. doi:10.3233/JAD-2011-111007 21897007

[21] Jack CR Jr, Bennett DA, Blennow K, . NIA-AA research framework: toward a biological definition of Alzheimer’s disease. Alzheimers Dement. 2018;14(4):535-562. doi:10.1016/j.jalz.2018.02.018 29653606 PMC5958625

[22] Jack CR Jr, Bennett DA, Blennow K, . A/T/N: an unbiased descriptive classification scheme for Alzheimer disease biomarkers. Neurology. 2016;87(5):539-547. doi:10.1212/WNL.0000000000002923 27371494 PMC4970664

[23] Milà-Alomà M, Shekari M, Salvadó G, ; ALFA study. Cognitively unimpaired individuals with a low burden of Aβ pathology have a distinct CSF biomarker profile. Alzheimers Res Ther. 2021;13(1):134. doi:10.1186/s13195-021-00863-y 34315519 PMC8314554

[24] Oeckl P, Halbgebauer S, Anderl-Straub S, ; Consortium for Frontotemporal Lobar Degeneration German. Glial fibrillary acidic protein in serum is increased in Alzheimer’s disease and correlates with cognitive impairment. J Alzheimers Dis. 2019;67(2):481-488. doi:10.3233/JAD-180325 30594925

[25] Verberk IMW, Thijssen E, Koelewijn J, . Combination of plasma amyloid beta_(1-42/1-40)_ and glial fibrillary acidic protein strongly associates with cerebral amyloid pathology. Alzheimers Res Ther. 2020;12(1):118. doi:10.1186/s13195-020-00682-7 32988409 PMC7523295

[26] Asken BM, Elahi FM, La Joie R, . Plasma glial fibrillary acidic protein levels differ along the spectra of amyloid burden and clinical disease stage. J Alzheimers Dis. 2020;78(1):265-276. doi:10.3233/JAD-200755 32986672 PMC7727314

[27] Elahi FM, Casaletto KB, La Joie R, . Plasma biomarkers of astrocytic and neuronal dysfunction in early- and late-onset Alzheimer’s disease. Alzheimers Dement. 2020;16(4):681-695. doi:10.1016/j.jalz.2019.09.004 31879236 PMC7138729

[28] Ishiki A, Kamada M, Kawamura Y, . Glial fibrillar acidic protein in the cerebrospinal fluid of Alzheimer’s disease, dementia with Lewy bodies, and frontotemporal lobar degeneration. J Neurochem. 2016;136(2):258-261. doi:10.1111/jnc.13399 26485083

[29] Fukuyama R, Izumoto T, Fushiki S. The cerebrospinal fluid level of glial fibrillary acidic protein is increased in cerebrospinal fluid from Alzheimer’s disease patients and correlates with severity of dementia. Eur Neurol. 2001;46(1):35-38. doi:10.1159/000050753 11455181

[30] Jesse S, Steinacker P, Cepek L, . Glial fibrillary acidic protein and protein S-100B: different concentration pattern of glial proteins in cerebrospinal fluid of patients with Alzheimer’s disease and Creutzfeldt-Jakob disease. J Alzheimers Dis. 2009;17(3):541-551. doi:10.3233/JAD-2009-1075 19433893

[31] Abu-Rumeileh S, Steinacker P, Polischi B, . CSF biomarkers of neuroinflammation in distinct forms and subtypes of neurodegenerative dementia. Alzheimers Res Ther. 2019;12(1):2. doi:10.1186/s13195-019-0562-4 31892365 PMC6937795

[32] Fontana IC, Zimmer AR, Rocha AS, . Amyloid-β oligomers in cellular models of Alzheimer’s disease. J Neurochem. 2020;155(4):348-369. doi:10.1111/jnc.15030 32320074

[33] Carter SF, Schöll M, Almkvist O, . Evidence for astrocytosis in prodromal Alzheimer disease provided by ^11^C-deuterium-L-deprenyl: a multitracer PET paradigm combining ^11^C-Pittsburgh compound B and ^18^F-FDG. J Nucl Med. 2012;53(1):37-46. doi:10.2967/jnumed.110.087031 22213821

[34] Schöll M, Carter SF, Westman E, . Early astrocytosis in autosomal dominant Alzheimer’s disease measured in vivo by multi-tracer positron emission tomography. Sci Rep. 2015;5(1):16404. doi:10.1038/srep16404 26553227 PMC4639762

[35] Rodriguez-Vieitez E, Ni R, Gulyás B, . Astrocytosis precedes amyloid plaque deposition in Alzheimer APPswe transgenic mouse brain: a correlative positron emission tomography and in vitro imaging study. Eur J Nucl Med Mol Imaging. 2015;42(7):1119-1132. doi:10.1007/s00259-015-3047-0 25893384 PMC4424277

[36] Alcolea D, Martínez-Lage P, Sánchez-Juan P, . Amyloid precursor protein metabolism and inflammation markers in preclinical Alzheimer disease. Neurology. 2015;85(7):626-633. doi:10.1212/WNL.0000000000001859 26180139

[37] Milà-Alomà M, Salvadó G, Gispert JD, ; ALFA study. Amyloid beta, tau, synaptic, neurodegeneration, and glial biomarkers in the preclinical stage of the Alzheimer’s continuum. Alzheimers Dement. 2020;16(10):1358-1371. doi:10.1002/alz.12131 32573951 PMC7586814

[38] Cairns NJ, Bigio EH, Mackenzie IRAA, ; Consortium for Frontotemporal Lobar Degeneration. Neuropathologic diagnostic and nosologic criteria for frontotemporal lobar degeneration: consensus of the Consortium for Frontotemporal Lobar Degeneration. Acta Neuropathol. 2007;114(1):5-22. doi:10.1007/s00401-007-0237-2 17579875 PMC2827877

[39] Kövari E. Neuropathological spectrum of frontal lobe dementias. Front Neurol Neurosci. 2009;24:49-159. doi:10.1159/000197894 19182473

[40] Nagele RG, D’Andrea MR, Lee H, Venkataraman V, Wang HY. Astrocytes accumulate Aβ42 and give rise to astrocytic amyloid plaques in Alzheimer disease brains. Brain Res. 2003;971(2):197-209. doi:10.1016/S0006-8993(03)02361-8 12706236

[41] Simpson JE, Ince PG, Lace G, ; MRC Cognitive Function and Ageing Neuropathology Study Group. Astrocyte phenotype in relation to Alzheimer-type pathology in the ageing brain. Neurobiol Aging. 2010;31(4):578-590. doi:10.1016/j.neurobiolaging.2008.05.015 18586353

[42] Muramori F, Kobayashi K, Nakamura I. A quantitative study of neurofibrillary tangles, senile plaques and astrocytes in the hippocampal subdivisions and entorhinal cortex in Alzheimer’s disease, normal controls and non-Alzheimer neuropsychiatric diseases. Psychiatry Clin Neurosci. 1998;52(6):593-599. doi:10.1111/j.1440-1819.1998.tb02706.x 9895207

[43] Pike CJ, Cummings BJ, Cotman CW. Early association of reactive astrocytes with senile plaques in Alzheimer’s disease. Exp Neurol. 1995;132(2):172-179. doi:10.1016/0014-4886(95)90022-5 7789457

[44] Benussi A, Ashton NJ, Karikari TK, . Serum glial fibrillary acidic protein (GFAP) is a marker of disease severity in frontotemporal lobar degeneration. J Alzheimers Dis. 2020;77(3):1129-1141. doi:10.3233/JAD-200608 32804092

[45] Sun M, Liu N, Xie Q, . A candidate biomarker of glial fibrillary acidic protein in CSF and blood in differentiating multiple sclerosis and its subtypes: a systematic review and meta-analysis. Mult Scler Relat Disord. 2021;51:102870. doi:10.1016/j.msard.2021.102870 33819724

[46] Papa L, Lewis LM, Falk JL, . Elevated levels of serum glial fibrillary acidic protein breakdown products in mild and moderate traumatic brain injury are associated with intracranial lesions and neurosurgical intervention. Ann Emerg Med. 2012;59(6):471-483. doi:10.1016/j.annemergmed.2011.08.021 22071014 PMC3830977

[47] Papa L, Brophy GM, Welch RD, . Time course and diagnostic accuracy of glial and neuronal blood biomarkers GFAP and UCH-L1 in a large cohort of trauma patients with and without mild traumatic brain injury. JAMA Neurol. 2016;73(5):551-560. doi:10.1001/jamaneurol.2016.0039 27018834 PMC8805143

[48] Nylén K, Öst M, Csajbok LZ, . Increased serum-GFAP in patients with severe traumatic brain injury is related to outcome. J Neurol Sci. 2006;240(1-2):85-91. doi:10.1016/j.jns.2005.09.007 16266720

[49] Nylén K, Csajbok LZ, Öst M, . Serum glial fibrillary acidic protein is related to focal brain injury and outcome after aneurysmal subarachnoid hemorrhage. Stroke. 2007;38(5):1489-1494. doi:10.1161/STROKEAHA.106.478362 17395862

[50] Larsson IM, Wallin E, Kristofferzon ML, Niessner M, Zetterberg H, Rubertsson S. Post-cardiac arrest serum levels of glial fibrillary acidic protein for predicting neurological outcome. Resuscitation. 2014;85(12):1654-1661. doi:10.1016/j.resuscitation.2014.09.007 25260722

[51] Sweeney MD, Sagare AP, Zlokovic BV. Blood-brain barrier breakdown in Alzheimer disease and other neurodegenerative disorders. Nat Rev Neurol. 2018;14(3):133-150. doi:10.1038/nrneurol.2017.188 29377008 PMC5829048

[52] Giannoni P, Badaut J, Dargazanli C, . The pericyte-glia interface at the blood-brain barrier. Clin Sci (Lond). 2018;132(3):361-374. doi:10.1042/CS20171634 29439117

[53] Iliff JJ, Wang M, Liao Y, . A paravascular pathway facilitates CSF flow through the brain parenchyma and the clearance of interstitial solutes, including amyloid β. Sci Transl Med. 2012;4(147):147ra111. doi:10.1126/scitranslmed.3003748 22896675 PMC3551275

[54] Louveau A, Smirnov I, Keyes TJ, . Structural and functional features of central nervous system lymphatic vessels. Nature. 2015;523(7560):337-341. doi:10.1038/nature14432 26030524 PMC4506234

[55] Ashton NJ, Suárez-Calvet M, Karikari TK, . Effects of pre-analytical procedures on blood biomarkers for Alzheimer’s pathophysiology, glial activation, and neurodegeneration. Alzheimers Dement (Amst). 2021;13(1):e12168. doi:10.1002/dad2.12168 34124336 PMC8171159

[56] Abdelhak A, Hottenrott T, Morenas-Rodríguez E, . Glial activation markers in CSF and serum from patients with primary progressive multiple sclerosis: potential of serum GFAP as disease severity marker? Front Neurol. 2019;10:280. doi:10.3389/fneur.2019.00280 30972011 PMC6443875

