# Supplementary material for: Differences Between Plasma and Cerebrospinal Fluid Glial Fibrillary Acidic Protein Levels Across the Alzheimer Disease Continuum
Source: JAMA Neurol. 2021 Oct 18;78(12):1–13. doi: 10.1001/jamaneurol.2021.3671 (PMC8524356; doi:10.1001/jamaneurol.2021.3671)
Supplement: Supplement 1. — eTable 1. Demographic Characteristics and Biomarker Levels of the Study Cohorts by Clinical and Biomarker-Defined Groups Including Other non-AD Dementia groups (TRIAD and Paris Cohorts) eTable 2. ROC Analysis to Discriminate Diagnostic Groups in the Paris Cohort eTable 3. ROC Analyses of the Plasma Biomarker Combinations to Discriminate Aβ-Positive From Aβ-Negative Individuals eFigure 1. Correlations Between Plasma and CSF GFAP eFigure 2. Biomarker Correlations eFigure 3. Plasma and CSF GFAP Group Comparisons (Including Other Dementias) eFigure 4. Plasma and CSF GFAP Group Comparisons in Early Amyloid Accumulators eFigure 5. Plasma and CSF GFAP Associations With Amyloid PET eFigure 6. Plasma and CSF GFAP Associations With Tau PET eFigure 7. Aβ as a Mediator of the Association Between p-Tau and Plasma GFAP eFigure 8. Plasma and CSF GFAP Associations With Neuroinflammation Biomarkers eMethods. eReferences. [file jamaneurol-e213671-s001.pdf]

Supplementary Online Content

Benedet AL, Milà-Alomà M, Vrillon A, et al; Translational Biomarkers in Aging and Dementia (TRIAD) study, Alzheimer's and Families (ALFA) study, and BioCogBank Paris Lariboisière cohort. Differences Between Plasma and Cerebrospinal Fluid Glial Fibrillary Acidic Protein Levels Across the Alzheimer Disease Continuum. *JAMA Neurol.* Published online October 18, 2021. doi:10.1001/jamaneurol.2021.3671

eTable 1. Demographic Characteristics and Biomarker Levels of the Study Cohorts by Clinical and Biomarker-Defined Groups Including Other non-AD Dementia groups (TRIAD and Paris Cohorts)
eTable 2. ROC Analysis to Discriminate Diagnostic Groups in the Paris Cohort
eTable 3. ROC Analyses of the Plasma Biomarker Combinations to Discriminate Aβ-positive From Aβ-negative Individuals
eFigure 1. Correlations Between Plasma and CSF GFAP
eFigure 2. Biomarker Correlations
eFigure 3. Plasma and CSF GFAP Group Comparisons (Including Other Dementias)
eFigure 4. Plasma and CSF GFAP Group Comparisons in Early Aβ Accumulators
eFigure 5. Plasma and CSF GFAP Associations With Aβ PET
eFigure 6. Plasma and CSF GFAP Associations With Tau PET
eFigure 7. Aβ as a Mediator of the Association Between p-tau181 and Plasma GFAP
eFigure 8. Plasma and CSF GFAP Associations With Neuroinflammation Biomarkers
eMethods.
eReferences.

This supplementary material has been provided by the authors to give readers additional information about their work.

eTable 1. Demographic Characteristics and Biomarker Levels of the Study Cohorts by Clinical and Biomarker-Defined Groups Including Other non-AD Dementia groups (TRIAD and Paris Cohorts)

	**TRIAD** (n = 312)								**BioCogBank Paris Lariboisière** (n = 212)						
	**Young CU** (n = 35)	**CU−** (n = 114)	**CU+** (n = 42)	**MCI+** (n = 39)	**AD dementia** (n = 45)	**Non-AD[a]** (n = 25)	**FTD** (n =12)	***P*-value**	**CU−** (n = 21)	**MCI+** (n = 42)	**AD dementia** (n = 76)	**Non-AD[b]** (n = 48)	**FTD** (n = 13)	**DLB** (n = 12)	***P*-value**
**Age,** years	23.1 (1.78)	69.9 (9.39)	74.1 (7.68)	71.2 (7.69)	66.1 (9.71)	70.8 (11.0)	62.4 (6.2)	<.001	64.4 (9.5)	72.4 (7.9)	72.2 (8.4)	66.6 (9.7)	65.2 (7.2)	65.3 (7.7)	<.001
**Female,** n (%)	22 (62.9)	73 (64.0)	29 (69.0)	21 (53.8)	21 (46.7)	11 (44.0)	8 (66.7)	.17	14 (66.7)	26 (61.9)	47 (61.8)	29 (60.4)	6 (46.2)	5 (38.5)	.66
**Education,** years	16.6 (1.5)	15.6 (3.9)	14.8 (3.2)	15.2 (3.2)	14.6 (3.6)	13.8 (3.9)	14.6 (4.1)	.03	11.2 (1.6)	10.7 (1.8)	9.7 (2.0)	10.7 (1.9)	11.9 (3.7)	14.6 (1.3)	<.001
***APOE* ε4 carriers,** n (%)	8 (22.9)	29 (26.9)	12 (28.6)	23 (62.2)	24 (55.8)	5 (22.7)	1 (8.3)	<.001	6 (28.6)	24 (57.1)	49 (64.5)	7 (14.6)	2 (15.4)	4 (33.3)	<.001
**MMSE**	30 (0)	29 (1.0)	29 (1.0)	28 (2.2)	19 (6.1)	27 (2.1)	24.3 (8.7)	<.001	27.4 (2.5)	23.5 (4.4)	19.3 (5.6)	24.6 (3.7)	23.5 (4.8)	24.2 (5.4)	<.001
**Centiloids**	-11.6 (6.60)	-3.12 (8.59)	52.5 (31.2)	91.1 (36.0)	91.8 (40.0)	1.10 (12.3)	-13.9 (10.1)	<.001	na	na	na	na	na	na	na
**CSF biomarkers**															
**Aβ42/40**	0.091 (0.006)	0.087 (0.017)	0.055 (0.015)	0.043 (0.010)	0.045 (0.011)	0.082 (0.026)	0.07 (0.02)	<.001	0.095 (0.007)	0.044 (0.009)	0.042 (0.009)	0.089 (0.012)	0.095 (0.011)	0.083 (0.015)	<.001
**p-tau181** (pg/ml)	22.6 (7.10)	36.2 (14.4)	59.3 (35.2)	89.4 (34.6)	99.9 (55.8)	59.7 (63.5)	25.9 (8.3)	<.001	32.8 (8.6)	93.0 (46.9)	115.4 (59.3)	37.7 (16.4)	37.2 (14.2)	32.3 (12.0)	<.001
**t-tau** (pg/ml)	195.3 (48.1)	311.0 (126.8)	396.4 (197.0)	539.4 (210.1)	659.6 (331.7)	448.4 (398.6)	298.0 (196.0)	<.001	243.1 (70.9)	587.6 (280.3)	732.6 (390.7)	305.6 (148.6)	442.8 (427.0)	232.2 (82.6)	<.001
**NfL** (pg/ml)	184.6 (57.7)	1132 (1038)	862.5 (268.7)	1127 (257.7)	1646 (965.0)	1783 (1663)	1400 (657.0)	<.001	889.3 (352.1)	1532 (643.4)	1695 (673.0)	1456 (1214)	3093 (1997)	966.0 (522.0)	<.001
**GFAP** (pg/ml)	4134 (1483)	12506 (5148)	15669 (6771)	17114 (5890)	16314 (8513)	14074 (7497)	8850 (3800)	<.001	2423 (2194)	4189 (3313)	4601 (3759)	2872 (2356)	2268 (1710)	1867 (979.0)	.08

© 2021 Benedet AL et al. *JAMA Neurology*.

Plasma biomarkers															
**NfL** (pg/ml)	6.5 (2.7)	22.1 (9.81)	27.9 (24.8)	25.7 (14.4)	33.6 (13.5)	28.6 (11.4)	27.8 (15.3)	<.001	13.13 (6.8)	24.2 (10.4)	24.4 (8.7)	21.2 (16.7)	40.5 (35.3)	18.0 (5.8)	<.01
**p-tau181** (pg/ml)	7.9 (3.6)	9.9 (4.4)	14.8 (11.0)	18.1 (8.1)	24.1 (9.6)	11.8 (12.3)	9.6 (6.2)	<.001	3.0 (1.8)	11.5 (6.2)	12.8 (3.6)	9.5 (6.7)	5.2 (4.7)	3.9 (2.2)	<.001
**GFAP** (pg/ml)	95.1 (62.1)	185.1 (93.5)	285.0 (142.6)	333.5 (153.6)	388.1 (152.8)	188.9 (105.9)	156.0 (146.0)	<.001	161.2 (67.1)	368.6 (158.5)	376.4 (179.6)	185.0 (96.0)	178.9 (108.7)	220.6 (118.7)	<.001

Abbreviations: Aβ42, amyloid-β 42; Aβ40, amyloid-β 40; AD, Alzheimer's Disease; CSF, cerebrospinal fluid; CU−, Aβ-negative cognitively unimpaired; CU+, Aβ-positive cognitively unimpaired; GFAP, glial fibrillary acidic protein, MCI+, Aβ-positive mild cognitive impairment; MMSE, Mini-Mental State Examination; na, not available; NfL, neurofilament light chain; p-tau181, tau phosphorylated at threonine 181; t-tau, total tau.

Data shown as mean (SD) or n (%), as appropriate. Within each cohort, we used *t*-test or one-way ANOVA to compare age, education years and MMSE between groups and Pearson's chi-square to compare sex and *APOE* ε4 frequencies between groups. Centiloids and fluid biomarkers levels were compared with a one-way ANCOVA adjusted by age and sex and followed by false discovery rate (FDR) multiple comparison correction. Aβ status for group definition was based on PET visual rating in the TRIAD cohort, and on CSF Aβ42/40 and BioCogBank Paris Lariboisière cohorts.

[a]Among the non-AD group there were 21 MCI with a negative Aβ PET visual read (MCI−) and 4 were clinically diagnosed as AD dementia but have a negative Aβ PET visual read.

[b] All non-AD subjects were MCI with normal CSF Aβ40/42 levels (MCI).

© 2021 Benedet AL et al. *JAMA Neurology*.

eTable 2. ROC Analysis to Discriminate Diagnostic Groups in the Paris Cohort

		AUC (95% CI)	
	**CU *vs* MCI+**	**CU *vs* AD dementia**	**MCI− *vs* MCI+**
**Plasma GFAP**	0.91 (0.83 - 0.98)	0.89 (0.82 - 0.96)	0.85 (0.77 - 0.93)
**CSF GFAP**	0.73 (0.59 - 0.88)	0.74 (0.61 - 0.87)	0.65 (0.53 - 0.77)
**Plasma p-tau181**	0.95 (0.91 - 1.00)	0.96 (0.92 - 0.99)	0.81 (0.72 - 0.90)
**Plasma NfL**	0.87 (0.77 - 0.97)	0.90 (0.81 - 0.99)	0.66 (0.54 - 0.78)

Abbreviations: AD, Alzheimer's disease; AUC, area under the curve; CI, confidence interval; CSF, cerebrospinal fluid; CU, cognitively unimpaired; GFAP, glial fibrillary acidic protein; MCI−, Aβ-negative mild cognitive impairment; MCI+, Aβ-positive mild cognitive impairment; NfL, neurofilament light chain; p-tau181, tau phosphorylated at threonine 181.

ROC analyses to test whether plasma GFAP discriminates between groups in BioCogBank Paris Lariboisière cohort. We also included CSF GFAP, plasma p-tau181 and plasma NfL for comparison.

© 2021 Benedet AL et al. *JAMA Neurology*.

eTable 3. ROC Analyses of the Plasma Biomarker Combinations to Discriminate Aβ-positive From Aβ-negative Individuals

	**AUC (95% CI) Aβ+ vs Aβ−**						
	**CSF Aβ42/40**			**Aβ PET** (VR)		**Aβ PET** (CL cut-off)	
	**TRIAD**	**ALFA+**	**Paris**	**TRIAD**	**ALFA+**	**TRIAD** (CL24)	**ALFA** (CL30)
**Plasma GFAP + Plasma p-tau181**	0.84 (0.78 - 0.90)	0.72 (0.66 - 0.78)	0.93 (0.89 - 0.97)	0.87 (0.84 - 0.91)	0.77 (0.89 - 0.97)	0.89 (0.85 - 0.92)	0.86 (0.78 - 0.94)
vs Plasma GFAP	0.82 (0.75 - 0.88)	0.69[a] (0.63 - 0.75)	0.86[a] (0.80 - 0.91)	0.85[a] (0.79 - 0.91)	0.75 (0.67 - 0.84)	0.83[a] (0.77 - 0.89)	0.82 (0.72 - 0.92)
vs Plasma p-tau181	0.78[a] (0.71 - 0.85)	0.67[a] (0.62 - 0.73)	0.87[a] (0.82 - 0.92)	0.77[a] (0.70 - 0.85)	0.67[a] (0.58 - 0.76)	0.79[a] (0.71 - 0.86)	0.76[a] (0.67 - 0.86)
**Plasma GFAP + Plasma NfL**	0.82 (0.75 - 0.88)	0.69 (0.63 - 0.76)	0.86 (0.81 - 0.92)	0.83 (0.78 - 0.88)	0.74 (0.66 - 0.83)	0.83 (0.79 - 0.88)	0.80 (0.70 - 0.91)
vs Plasma GFAP	0.82 (0.75 - 0.88)	0.69 (0.63 - 0.75)	0.86 (0.80 - 0.91)	0.85 (0.79 - 0.91)	0.75 (0.67 - 0.84)	0.83 (0.77 - 0.89)	0.82 (0.72 - 0.92)
vs Plasma NfL	0.74 (0.67 - 0.82)	0.63[b] (0.57 - 0.69)	0.74[b] (0.65 - 0.83)	0.67[b] (0.59 - 0.76)	0.66 (0.58 - 0.75)	0.68[b] (0.59 - 0.76)	0.73 (0.63 - 0.83)
**Plasma GFAP + Plasma p-tau181 + Plasma NfL**	0.84 (0.78 - 0.90)	0.72 (0.66 - 0.79)	0.93 (0.90 - 0.97)	0.88 (0.84 - 0.91)	0.77 (0.68 - 0.85)	0.89 (0.85 - 0.92)	0.86 (0.77 - 0.94)
vs Plasma GFAP	0.82 (0.75 - 0.88)	0.69[c] (0.63 - 0.75)	0.86[c] (0.80 - 0.91)	0.85[d] (0.79 - 0.91)	0.75 (0.67 - 0.84)	0.83[d] (0.77 - 0.89)	0.82 (0.72 - 0.92)
vs Plasma p-tau181	0.78[c] (0.71 - 0.85)	0.67[c] (0.62 - 0.73)	0.87[c] (0.82 - 0.92)	0.77[c] (0.70 - 0.85)	0.67[c] (0.58 - 0.76)	0.79[c] (0.71 - 0.86)	0.76[c] (0.67 - 0.86)
vs Plasma NfL	0.74[c] (0.67 - 0.82)	0.63[d] (0.57 - 0.69)	0.74[d] (0.65 - 0.83)	0.67[d] (0.59 - 0.76)	0.66[c] (0.58 - 0.75)	0.68[d] (0.59 - 0.76)	0.73[c] (0.63 - 0.83)

Abbreviations: Aβ, amyloid-β; AUC, area under the curve; CI, confidence interval; CL, Centiloid; CSF, cerebrospinal fluid; GFAP, glial fibrillary acidic protein; NfL, neurofilament light chain; p-tau181, tau phosphorylated at threonine 181; PET, positron emission tomography; VR, visual read.

ROC analyses to test whether the combination of plasma GFAP with the other plasma biomarkers discriminate between Aβ-positive (Aβ+) and Aβ-negative individuals (Aβ−), as defined by the CSF Aβ42/40 ratio, Aβ PET visual read or Aβ PET using a cut-off of 24 (TRIAD) or 30 (ALFA) Centiloids. We compared each biomarker combination with their individual biomarkers. AUCs differences were tested using the DeLong test followed by FDR multiple comparison correction. The significant differences were as follows:

[a]*P* < .05 *vs* plasma GFAP + plasma p-tau181
[b]*P* < .05 *vs* plasma GFAP + plasma NfL
[c]*P* < .05 *vs* plasma GFAP + plasma p-tau181 + plasma NfL
[d]*P* < .01 *vs* plasma GFAP + plasma p-tau181 + plasma NfL

© 2021 Benedet AL et al. *JAMA Neurology*.

eFigure 1. Correlations Between Plasma and CSF GFAP

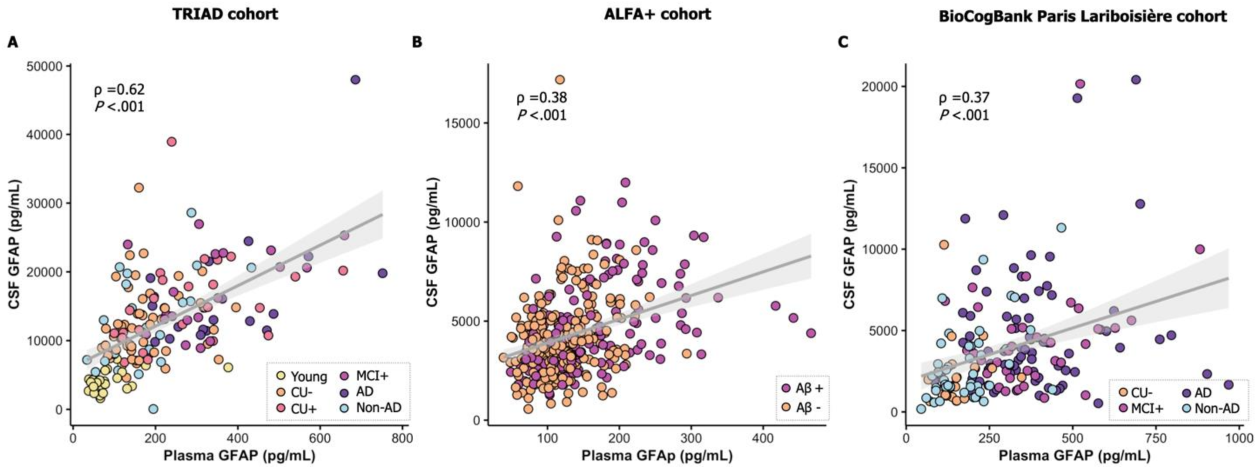

A-C Scatter plots depicting the correlations between CSF and plasma GFAP in TRIAD (A), ALFA+ (B) and BioCogBank Paris Lariboisière (C) cohorts. Individuals are coloured by clinical diagnosis (TRIAD and BioCogBank Paris Lariboisière cohorts) or Aβ status (ALFA+) group. Correlations were assessed with Spearman rank test correlation coefficient (*ρ*) and *P* values are shown for each cohort. Aβ-positivity was defined with Aβ PET visual read in TRIAD, and with CSF Aβ42/40 in ALFA+ and BioCogBank Paris Lariboisière cohorts. The solid lines indicate the regression line and the 95% confidence intervals.

Abbreviations: Aβ, amyloid-β; AD, Alzheimer's disease; CSF, cerebrospinal fluid; CU−, Aβ-negative cognitively unimpaired; CU+, Aβ-positive cognitively unimpaired; GFAP, glial fibrillary acidic protein, MCI+, Aβ-positive mild cognitive impairment.

© 2021 Benedet AL et al. *JAMA Neurology*.

eFigure 2. Biomarker Correlations

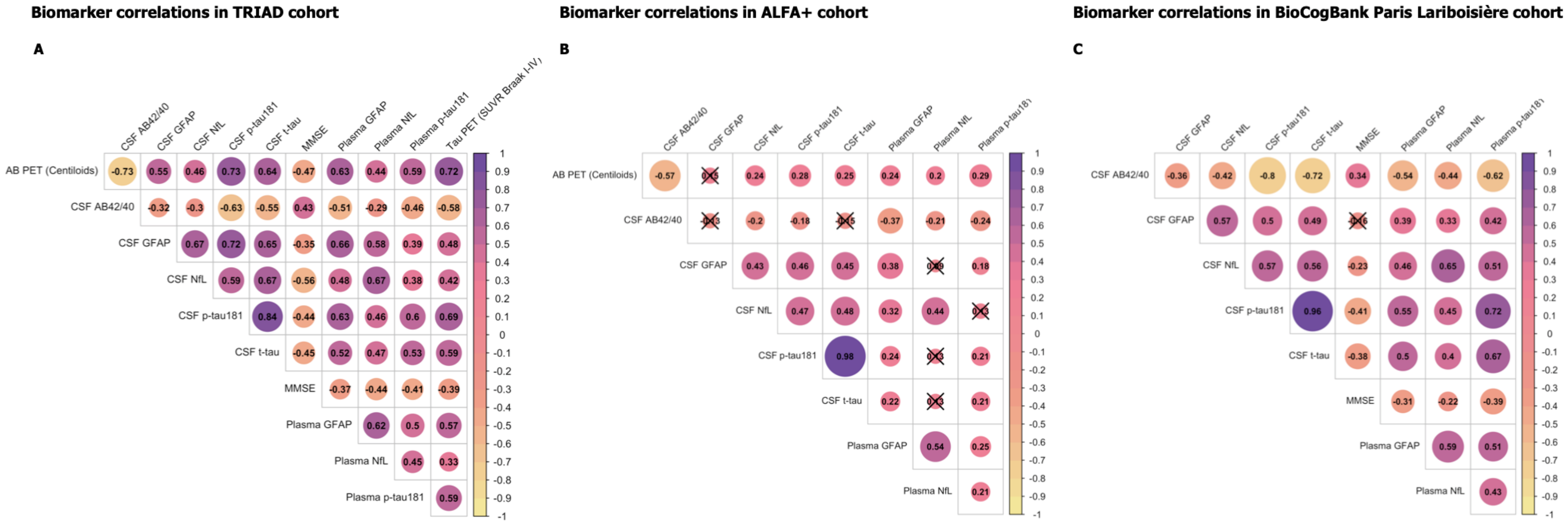

A-C Correlations between biomarkers in the TRIAD (panel A), ALFA+ (panel B) and BioCogBank Paris Lariboisière (Panel C) cohorts. Purple colour indicates a positive correlation and yellow colour a negative correlation. Colour intensity and the size of the circle are proportional to the Spearman correlation coefficients (ρ). Crossed cells indicate non-significant correlations.

Abbreviations: Aβ, amyloid-β; GFAP, glial fibrillary acidic protein, MMSE, Mini-Mental State Examination; NfL, neurofilament light chain; p-tau181, tau phosphorylated at threonine 181, t-tau, total tau.

© 2021 Benedet AL et al. *JAMA Neurology*.

eFigure 3. Plasma and CSF GFAP Group Comparisons (Including Other Dementias)

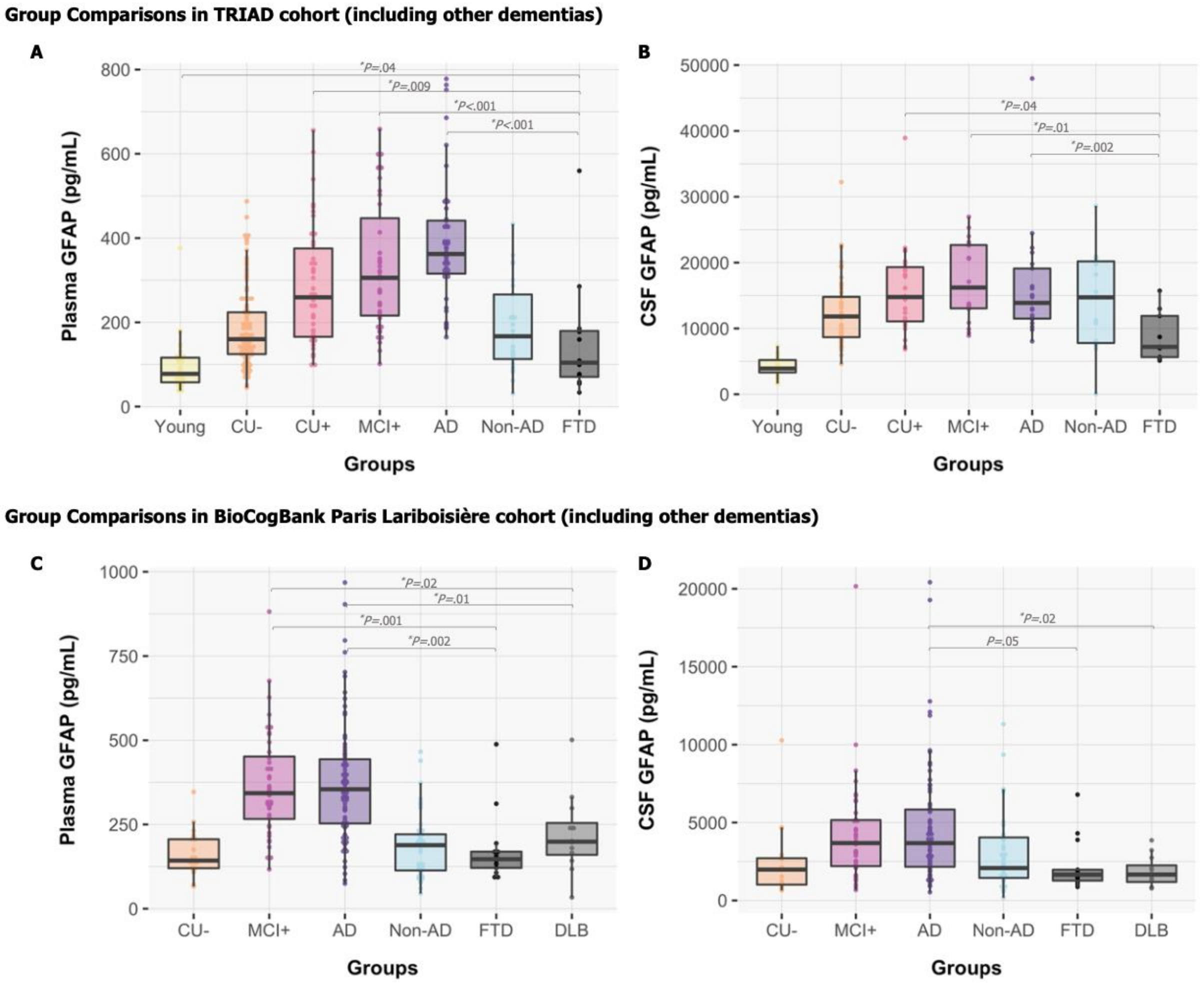

A-D Box-plots comparing CSF and plasma GFAP levels across groups in TRIAD (panels A, B), and BioCogBank Paris Lariboisière cohort (panels C, D) including other non-AD dementias. The box-plots depict the median (horizontal bar), interquartile range (IQR, hinges) and 1.5 x IQR (whiskers). Group comparisons were computed with a one-way ANCOVA adjusting for age and sex. Tukey honestly significant difference (HSD) test was used for the post hoc pairwise comparisons in all cohorts.

Abbreviations: Aβ, amyloid-β; AD, Alzheimer's disease; CSF, cerebrospinal fluid; CU−, Aβ-negative cognitively unimpaired; CU+, Aβ-positive cognitively unimpaired; DLB, dementia with Lewy bodies; FTD, frontotemporal dementia, GFAP, glial fibrillary acidic protein, MCI+, Aβ-positive mild cognitive impairment.

© 2021 Benedet AL et al. *JAMA Neurology*.

eFigure 4. Plasma and CSF GFAP Group Comparisons in Early Aβ Accumulators

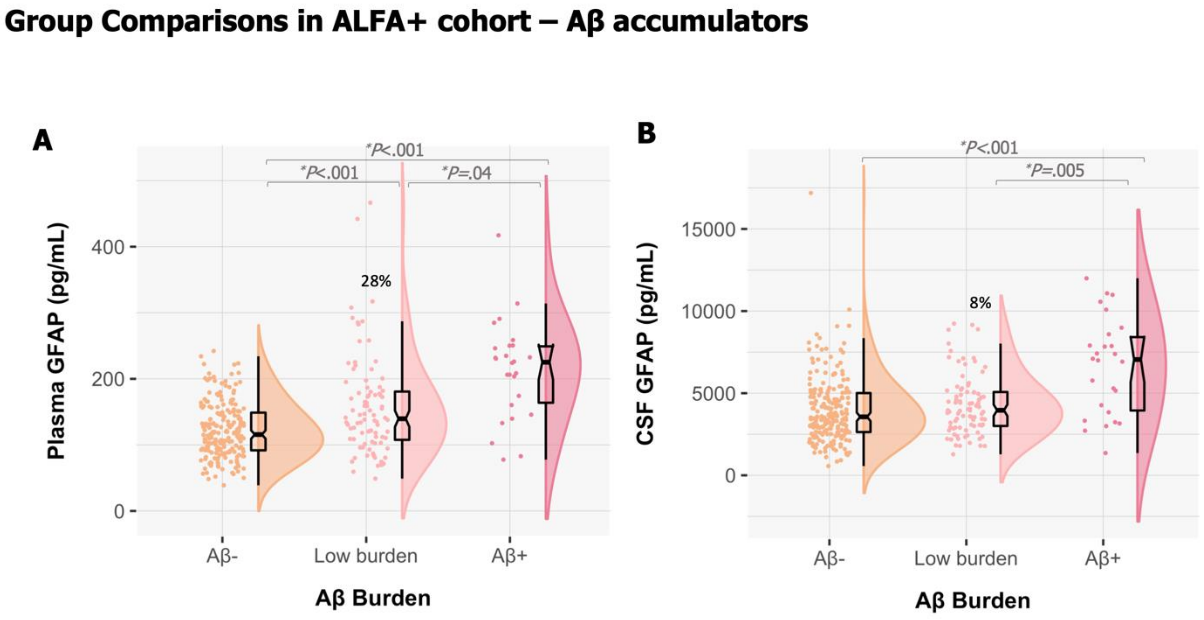

A, B Violin plots comparing plasma and CSF GFAP levels in ALFA+ individuals by Aβ burden groups. Aβ burden groups were defined by a combination of CSF Aβ42/40 and Aβ PET. This classification comprises the following 3 groups: (i) Aβ-negative (Aβ−; negative CSF Aβ42/40 and Aβ PET < 30 Centiloids), (ii) group with low burden of Aβ pathology (Low burden; positive CSF Aβ42/40 but Aβ PET < 30 Centiloids), and (iii) Aβ-positive (Aβ+; positive CSF Aβ42/40 and Aβ PET ≥ 30 Centiloids). Group comparisons were computed with a one-way ANCOVA adjusting for age and sex, followed by Tukey-corrected post hoc pairwise comparisons. Fold changes were calculated using Aβ-negative as reference group.

Abbreviations: Aβ, amyloid-β; CSF, cerebrospinal fluid; GFAP, glial fibrillary acidic protein.

© 2021 Benedet AL et al. *JAMA Neurology*.

eFigure 5. Plasma and CSF GFAP Associations With Aβ PET

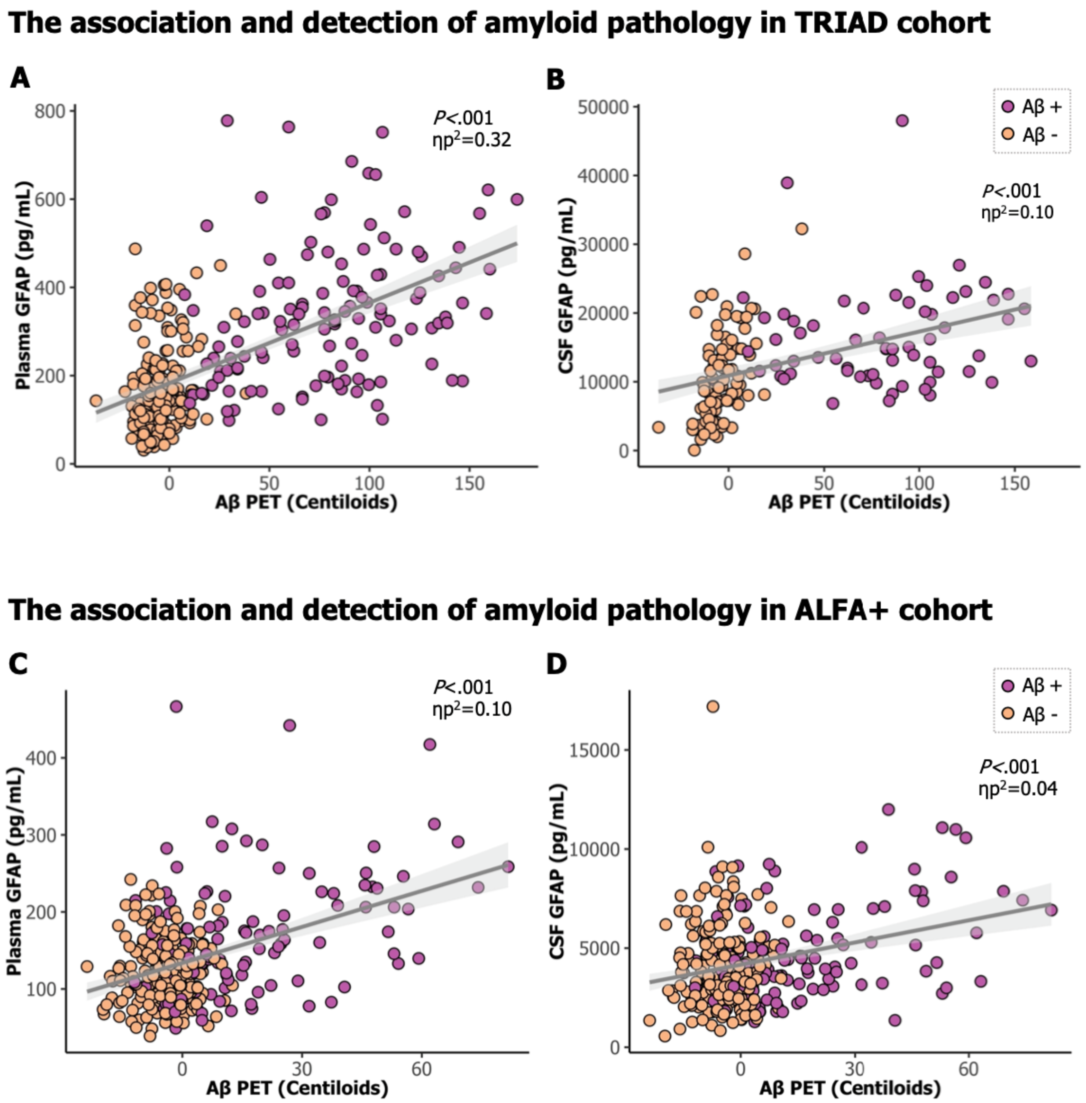

Scatter plots depicting the associations between plasma and CSF GFAP with Aβ PET Centiloids in the TRIAD (panels A, B) and ALFA+ (panels C, D) cohorts. Individuals are coloured by Aβ status (as defined by Aβ PET visual read in TRIAD and CSF Aβ42/40 ratio in ALFA+). The solid lines indicate the regression line and the 95% confidence intervals. *P* values were computed with linear models adjusted by age, sex and clinical diagnosis (the latter only for TRIAD cohort). The sizes of the associations between variables are shown by the partial eta-squared (η_p^2).

Abbreviations: Aβ, amyloid-β; CSF, cerebrospinal fluid; GFAP, glial fibrillary acidic protein; PET, positron emission tomography.

© 2021 Benedet AL et al. *JAMA Neurology*.

eFigure 6. Plasma and CSF GFAP Associations With Tau PET

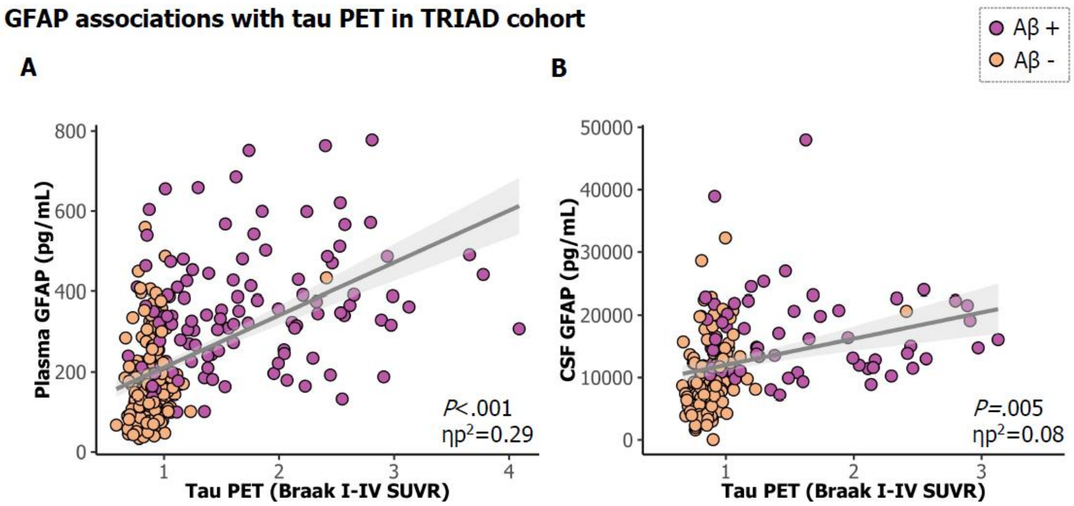

A,B Scatter plots depicting the associations between plasma and CSF GFAP with tau PET in the TRIAD cohort. Individuals are coloured by Aβ status (as defined by Aβ PET visual read). The solid lines indicate the regression line and the 95% confidence intervals. *P* values were computed with linear models adjusted by age, sex and clinical diagnosis. The sizes of the associations between variables are shown by the partial eta-squared (η_p^2).

Abbreviations: Aβ, amyloid-β; CSF, cerebrospinal fluid; GFAP, glial fibrillary acidic protein; PET, positron emission tomography.

© 2021 Benedet AL et al. *JAMA Neurology*.

eFigure 7. Aβ as a Mediator of the Association Between p-tau181 and Plasma GFAP

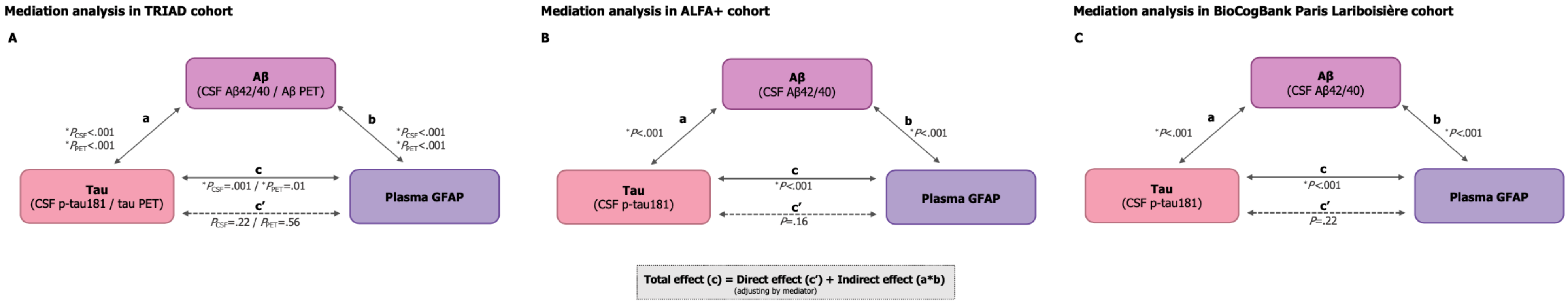

A-C Mediation analysis of the association between CSF p-tau181 or tau PET and plasma GFAP in the TRIAD (panel A), ALFA+ (panel B) and BioCogBank Paris Lariboisière (panel C) cohorts. *P* values for the analysis with CSF Aβ42/40 as mediator and CSF p-tau181 as independent variable are shown. In TRIAD, *P* values are also shown for analyses with Aβ PET as mediator and tau PET as independent variable. The direct effect of tau on the mediator is *a*, and the direct effect of the mediator on plasma GFAP is *b*. The indirect effect is designated *a**b* whilst the direct effect of tau on plasma GFAP after adjusting for the mediator is c'. The total effect of tau on plasma GFAP (c) is designated as the summation of the direct effect adjusted by the mediator(*c'*) and the indirect effect *(a**b)*.

Abbreviations: Aβ, amyloid-β; CSF, cerebrospinal fluid; GFAP, glial fibrillary acidic protein; PET, positron emission tomography; p-tau181, tau phosphorylated at threonine 181.

© 2021 Benedet AL et al. *JAMA Neurology*.

eFigure 8. Plasma and CSF GFAP Associations With Neuroinflammation Biomarkers

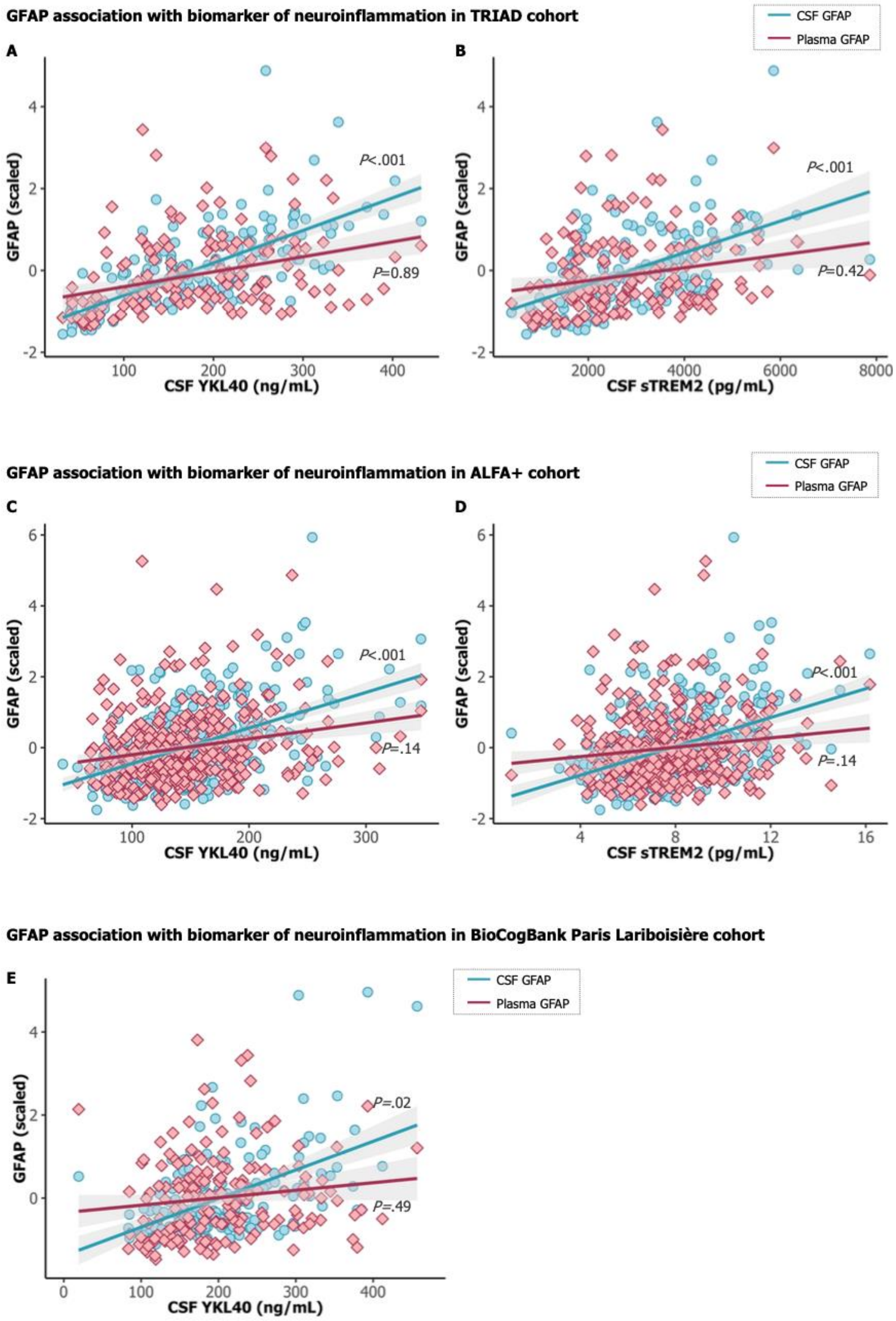

A-E Scatter plots depicting the association of plasma (red) and CSF (blue) GFAP with glial markers CSF YKL40 and CSF sTREM2 in the TRIAD and ALFA+ cohorts and with CSF YKL40 in the BioCogBank Paris

© 2021 Benedet AL et al. *JAMA Neurology*.

Lariboisière cohort. CSF sTREM2 was not available for the BioCogBank Paris Lariboisière cohort. The solid lines indicate the regression line and the 95% confidence intervals. *P* values were computed with linear models adjusted by age, sex and clinical diagnosis (the latter only for TRIAD and BioCogBank Paris Lariboisière cohorts). Plasma and CSF GFAP values were scaled.

Abbreviations: CSF, cerebrospinal fluid; GFAP, glial fibrillary acidic protein; sTREM2, soluble triggering receptor expressed on myeloid cells 2; YKL40, chitinase-3-like protein 1.

© 2021 Benedet AL et al. *JAMA Neurology*.

eMethods.

Study population

This cross-sectional study included participants from three cohorts: (a) the Translational Biomarkers in Aging and Dementia (TRIAD) cohort,[1] (b) the ALFA+ cohort,[2] and (c) the BioCogBank Paris Lariboisière cohort (a clinical cohort).[3]

TRIAD is a longitudinal and biomarker-based cohort, with yearly assessments and evaluation of cognitive status, fluid sample collection and imaging data acquisition. TRIAD includes participants across the full Alzheimer disease (AD) spectrum, from cognitively unimpaired (CU) young adults, to CU elderlies, mild cognitive impairment (MCI) and finally AD dementia patients. The AD dementia diagnosis was given following the National Institute on Aging and the Alzheimer's Association criteria for probable AD,[4] with a Clinical Dementia Rating (CDR) greater than 1. CU individuals had CDR of 0, whilst MCI patients had CDR of 0.5, subjective and objective memory impairments, and essentially normal activities of daily living. We included in this particular study only participants who had plasma biomarkers and Aβ imaging data, that is a total of 300 subjects (35 young, 156 CU, 60 MCI and 49 dementia). A fraction of them also had CSF measurements (n = 159, 53%) or tau PET data (n = 293, 97%). For completeness, 12 Frontotemporal dementia (FTD) patients were also studied in an additional analysis (eFigure 3). These had a clinical diagnosis of the behavioural or semantic variant of FTD, a CDR score > 0.5, and were Aβ PET-negative. For the purposes of this study, participants were further classified according to both clinical diagnosis (CU, MCI and dementia) and Aβ status (based on Aβ PET visual rating) as Aβ-positive (Aβ+) or Aβ-negative (Aβ−). The resulting clinical and biomarker-defined groups were: 35 young CU, 114 CU−, 42 CU+, 39 MCI+, 45 AD dementia

and 25 non-AD cognitively impaired (21 MCI− and 4 clinically diagnosed as AD Dementia syndrome but Aβ−).

The ALFA+ cohort is a nested longitudinal study from the ALFA (for Alzheimer's and Families) study.[2] The ALFA study includes 2,743 cognitively unimpaired (CDR = 0; MMSE ≥ 26; semantic fluency ≥ 12), middle-aged individuals at elevated risk for AD, with a high proportion of AD patients' offspring and *APOE* ε4 carriership. The ALFA+ cohort includes ~400 participants, who are widely phenotyped with extensive clinical and neuropsychological assessments, and includes acquisition of fluid (CSF and blood) and imaging (magnetic resonance imaging [MRI] and PET) biomarkers. Among the 400 ALFA+ participants, 397 had available CSF biomarkers. In ALFA+, Aβ status was defined by CSF Aβ42/40 (Aβ-positive if CSF Aβ42/40 < 0.071 and Aβ-negative if CSF Aβ42/40 ≥ 0.071). In addition, for the AT classification, participants were classified as tau-positive if CSF p-tau181 > 24 pg/ml.[5] There were 13 participants who were Aβ-negative but p-tau-positive (and, therefore, not within the AD continuum); these participants were included in the initial comparison between AT groups, but excluded in the rest of the analyses, which focused on the AD continuum. Thus, a total of 384 participants were included in the study, of whom 327 (85.2%) also had available Aβ PET. Moreover, in order to study the earliest stages of preclinical AD, we also used a previously described Aβ burden classification based on the combination of CSF Aβ42/40 and Aβ PET[6]. This classification comprises the following 3 groups: (i) Aβ-negative (negative CSF Aβ42/40 and Aβ PET < 30 Centiloids), ii) group with low burden of Aβ pathology (positive CSF Aβ42/40 but Aβ PET < 30 Centiloids), and iii) Aβ-positive (positive CSF Aβ42/40 and Aβ PET ≥ 30 Centiloids).

The BioCogBank Paris Lariboisière cohort was issued by the Center of Cognitive Neurology, University Hospital Lariboisière Fernand Widal. This department specializes in

© 2021 Benedet AL et al. *JAMA Neurology*.

management of patients with cognitive disorders and neurodegenerative diseases. Individuals who underwent neuropsychological assessment, MRI and CSF analysis in the center for a neurocognitive complaint were included. Consensus clinical diagnosis was reached after discussion between neurologists, geriatricians and neuropsychologists, according to actual diagnostic criteria for AD and MCI.[7] AD dementia and MCI+ subjects had a CSF biomarker profile on the AD continuum with abnormal CSF Aβ40/42 ratio (CSF Aβ42/40 cut-off for Aβ positivity was established at 0.068), whilst the non-AD group included MCI−, that is individuals with the clinical diagnosis of MCI but with normal CSF Aβ42/40 levels. Control participants consulted because of a cognitive complain or were included in observational research studies. They were classified as CU when a diagnostic of neurocognitive disorder was excluded by the neurologist in charge and, additionally, they fulfilled the following conditions: (i) the neuropsychological assessment found preserved global cognition (*i.e.* normative or subnormative scores for age, sex and level of education), (ii) brain MRI did not find any sign of atrophy, (iii) they displayed a normal CSF biomarker profile and (iv) no cognitive decline was observed during follow-up. In addition, 13 FTD and 12 Dementia with Lewy-bodies (DLB) subjects were also studied in an additional analysis (eFigure 3). Patients with FTD included patients with behavioural variant and semantic dementia.[8,9] They displayed a normal CSF Aβ42/40 ratio. Patients with DLB were diagnosed according recent clinical criteria.[10,11] As in the TRIAD cohort, participants with clinical non-AD dementia were excluded in the subsequent analyses. Thus, this cohort included 187 participants: 21 CU−, 42 MCI+, 76 AD dementia and 48 non-AD (all MCI−).

All studies have been approved by their regional ethical committees and all study participants provided written informed consent.

© 2021 Benedet AL et al. *JAMA Neurology*.

CSF and plasma collection and biomarkers measurements

Plasma and CSF samples from the three cohorts were independently analysed at the Clinical Neurochemistry Laboratory at the University of Gothenburg, Sweden. For all three cohorts, we used a Simoa HD-X instrument (Quanterix, Billerica, MA, USA) to quantify plasma and CSF GFAP (using the commercial single-plex assay #102336), plasma p-tau181 (using an *in-house* immunoassay previously described)[12], and plasma NfL (using an *in-house* immunoassay previously described[13] for TRIAD, or using the commercial single-plex assay NF-light Kit #103186 for ALFA+ and Paris cohorts).

For TRIAD, CSF and plasma collection followed procedures previously described.[12] CSF Aβ40, Aβ42, p-tau181 and t-tau were quantified with the LUMIPULSE G1200 (Fujirebio). CSF sTREM2 was measured using an *in-house* electrochemiluminescence assay on the MesoScale Discovery SECTOR imager 6000 (MesoScale Discovery (MSD), Maryland, USA),[14,15] while CSF YKL40 was quantified with a commercial ELISA assay (R&D Systems, Minneapolis, MN). CSF Aβ42/40 cut-off for positivity was 0.068.

In ALFA+, CSF samples were obtained by lumbar puncture following standard procedures.[5,16] Blood samples collection and processing procedure was previously described.[17] CSF p-tau181 and t-tau were measured using the electrochemiluminescence Elecsys® Phospho-Tau (181P) CSF and Total-Tau CSF immunoassays on a fully automated cobas e 601 module (Roche Diagnostics International Ltd). CSF Aβ40, Aβ42, NfL, sTREM2 and YKL40 were measured with the exploratory NTK robust prototype immunoassays (Roche Diagnostic International Ltd) on a cobas e 411 analyzer or cobas e 601 module.

In the BioCogBank Paris Lariboisière cohort, venous and lumbar punctures were performed after an overnight fast. CSF was collected for analysis, centrifuged at 4°C, immediately

© 2021 Benedet AL et al. *JAMA Neurology*.

aliquoted, and frozen at −80°C until assayed. Plasma was centrifuged at 2000g for 20 min at 4°C. Plasma supernatant was collected and frozen at −80°C until further use. CSF biomarkers were assayed using Lumipulse G1200 (Fujirebio). CSF YKL40 concentration was measured using a commercially available ELISA kit (R&D Systems, Minneapolis, MN).

Imaging biomarkers

In TRIAD, Aβ PET was quantified with [^{18}F]AZD4694 (40–70 minutes post-injection) and tau PET was quantified with [^{18}F]MK6240 (90–110 minutes post-injection). PET scans were acquired with a Siemens High Resolution Research Tomograph (Siemens Medical Solutions, Knoxville, TN) and imaging data were processed as previously described.[1,18] Aβ status was assessed based on visual reading of [^{18}F]AZD4694 PET by two neurologists blinded to clinical diagnosis. In addition, Aβ PET SUVR was also converted to Centiloid units[19] as previously described.[18,20] The PET SUVR cut-off value of 1.55 corresponds to 24 Centiloids. The MRI T1-weighted images were acquired at 3T following an initial preprocessing with intensity normalization and gradient unwarping. Images were then processed using DARTEL and registered using a six-parameter affine transformation and nonlinearly spatially normalized to the ADNI template.[21]

In ALFA+, [^{18}F]flutemetamol (Aβ) PET scans acquisition and pre-processing procedures have been previously described.[22] In brief, [^{18}F] flutemetamol (Aβ) PET scans were acquired on a Biograph mCT scanner (Siemens Healthcare, Erlangen, Germany) following a cranial CT scan for attenuation correction. Participants were injected with 185 MBq (range 166.5– 203.5 Mbq) of [^{18}F] flutemetamol, and four frames of 5 min each were acquired 90 min post-injection. [^{18}F]flutemetamol PET processing was performed following a validated Centiloid pipeline[19] using

© 2021 Benedet AL et al. *JAMA Neurology*.

SPM12.[22] Centiloid values were calculated from the mean values of the standard Centiloid target region (http://www.gaain.org/centiloid-project) using the transformation previously calibrated.[22] Aβ PET images were visually rated by a nuclear medicine physician as Aβ+ or Aβ- using standard clinical criteria as specified in the Summary of Product Characteristics of the tracer.[23] MRI scans were obtained with a 3T scanner (Ingenia CX, Philips, Amsterdam, Netherlands). The MRI protocol was identical for all participants and included a high-resolution 3D T1-weighted Turbo Field Echo (TFE) sequence (voxel size 0.75 x 0.75 x 0.75 mm, TR/TE: 9.90/4.6 ms, flip angle = 8º). T1-weighted images were automatically segmented using FreeSurfer 6.0. Segmentation results were visually quality-controlled by an expert.

Statistical analysis

We tested for normality of the distribution for each biomarker using visual inspection of histograms, and those variables that did not follow a normal distribution were log10-transformed. We used linear regression models to assess the association between plasma or CSF GFAP and the other biomarkers, adjusting by age and sex and, in TRIAD and BioCogBank Paris Lariboisière cohorts, also for clinical diagnosis. In these models, all quantitative variables were scaled and, when relevant, partial eta-squared (η_p^2) was reported as a measure of the effect size. Similar models were also applied to evaluate group differences, as well as age and sex effects, and, when necessary, Tukey honestly significant difference (HSD) test was used for the post hoc pairwise comparisons in all cohorts. Fold changes were calculated using CU− as reference group and the effect size of the difference between groups was estimated by calculating Cohen's *d* (*d*), in which the dependent variable was the residuals of log-transformed GFAP (plasma or CSF) regressed on age and sex. When correlations were evaluated, Spearman rank test was employed using raw

© 2021 Benedet AL et al. *JAMA Neurology*.

biomarker values. Receiver operating curves (ROC) provided both the area under the curve (AUC) for Aβ positivity, or diagnostic groups, and the representative best value for accuracy at an optimal cut-off value. AUCs for the different biomarkers were compared using the "*pROC"* package and False discovery rate (FDR) was used to correct *P* values for multiple comparisons. Finally, mediation analysis was performed with the R package "*mediation*" and standardized indirect effects were computed for each 1000 bootstrapped samples. 95% confidence intervals were computed by calculating the indirect effects at 2.5th and 97.5th percentiles. For these analyses, plasma and CSF GFAP were, separately, the outcome variable and Aβ and tau biomarkers the predictor variables, adjusting for age, sex and diagnosis.

All tests were 2-tailed, with a significance level of $\alpha = 0.05$. All statistical analyses were performed and figures built in R software (version 3.6.3).

© 2021 Benedet AL et al. *JAMA Neurology*.

eReferences.

1. Pascoal TA, Shin M, Kang MS, et al. In vivo quantification of neurofibrillary tangles with [18 F]MK-6240. *Alzheimer's Res Ther*. 2018;10(1). doi:10.1186/s13195-018-0402-y
2. Molinuevo JL, Gramunt N, Gispert JD, et al. The ALFA project: A research platform to identify early pathophysiological features of Alzheimer's disease. *Alzheimer's Dement Transl Res Clin Interv*. 2016;2(2):82-92. doi:10.1016/j.trci.2016.02.003
3. Dumurgier J, Paquet C, Peoc'H K, et al. CSF Aβ1-42 levels and glucose metabolism in Alzheimer's disease. *J Alzheimer's Dis*. 2011;27(4):845-851. doi:10.3233/JAD-2011-111007
4. McKhann GM, Knopman DS, Chertkow H, et al. The diagnosis of dementia due to Alzheimer's disease: Recommendations from the National Institute on Aging-Alzheimer's Association workgroups on diagnostic guidelines for Alzheimer's disease. *Alzheimer's Dement*. 2011;7(3):263-269. doi:10.1016/j.jalz.2011.03.005
5. Milà-Alomà M, Salvadó G, Gispert JD, et al. Amyloid beta, tau, synaptic, neurodegeneration, and glial biomarkers in the preclinical stage of the Alzheimer's continuum. *Alzheimer's Dement*. 2020;16(10):1358-1371. doi:10.1002/alz.12131
6. Milà-Alomà M, Shekari M, Salvadó G, et al. Cognitively unimpaired individuals with a low burden of Aβ pathology have a distinct CSF biomarker profile. *Alzheimers Res Ther*. 2021;13(1):134. doi:10.1186/s13195-021-00863-y
7. Jack CR, Bennett DA, Blennow K, et al. NIA-AA Research Framework: Toward a biological definition of Alzheimer's disease. *Alzheimer's Dement*. 2018;14(4):535-562. doi:10.1016/j.jalz.2018.02.018
8. Rascovsky K, Hodges JR, Knopman D, et al. Sensitivity of revised diagnostic criteria for the behavioural variant of frontotemporal dementia. *Brain*. 2011;134(9):2456-2477. doi:10.1093/brain/awr179
9. Gorno-Tempini ML, Hillis AE, Weintraub S, et al. Classification of primary progressive aphasia and its variants. *Neurology*. 2011;76(11):1006-1014. doi:10.1212/WNL.0b013e31821103e6
10. McKeith IG, Ferman TJ, Thomas AJ, et al. Research criteria for the diagnosis of prodromal dementia with Lewy bodies. *Neurology*. 2020;94(17):743-755. doi:10.1212/WNL.0000000000009323
11. McKeith IG, Boeve BF, Dickson DW, et al. Diagnosis and management of dementia with Lewy bodies. *Neurology*. 2017;89(1):88-100. doi:10.1212/WNL.0000000000004058
12. Karikari TK, Pascoal TA, Ashton NJ, et al. Blood phosphorylated tau 181 as a biomarker for Alzheimer's disease: a diagnostic performance and prediction modelling study using data from four prospective cohorts. *Lancet Neurol*. 2020;19(5):422-433. doi:10.1016/S1474-4422(20)30071-5
13. Gisslén M, Price RW, Andreasson U, et al. Plasma Concentration of the Neurofilament Light Protein (NFL) is a Biomarker of CNS Injury in HIV Infection: A Cross-Sectional Study. *EBioMedicine*. 2016;3:135-140. doi:10.1016/j.ebiom.2015.11.036
14. Suárez-Calvet M, Kleinberger G, Araque Caballero MÁ, et al. sTREM cerebrospinal fluid levels are a potential biomarker for microglia activity in early-stage Alzheimer's disease and associate with neuronal injury markers. *EMBO Mol Med*. 2016;8(5):466-476. doi:10.15252/emmm.201506123
15. Kleinberger G, Yamanishi Y, Suarez-Calvet M, et al. TREM2 mutations implicated in neurodegeneration impair cell surface transport and phagocytosis. *Sci Transl Med*. 2014;6(243):243ra86-243ra86. doi:10.1126/scitranslmed.3009093
16. Teunissen CE, Tumani H, Engelborghs S, Mollenhauer B. Biobanking of CSF: International standardization to optimize biomarker development. *Clin Biochem*. 2014;47(4-5):288-292. doi:10.1016/j.clinbiochem.2013.12.024
17. Suárez-Calvet M, Karikari TK, Ashton NJ, et al. Novel tau biomarkers phosphorylated at T181, T217 or T231 rise in the initial stages of the preclinical Alzheimer's continuum when only subtle changes in Aβ pathology are detected . *EMBO Mol Med*. 2020;12(12):1-19. doi:10.15252/emmm.202012921
18. Therriault J, Benedet AL, Pascoal TA, et al. Determining Amyloid-β Positivity Using 18F-AZD4694 PET Imaging. *J Nucl Med*. 2021;62(2):247-252. doi:10.2967/jnumed.120.245209
19. Klunk WE, Koeppe RA, Price JC, et al. The Centiloid Project: Standardizing quantitative amyloid plaque estimation by PET. *Alzheimer's Dement*. 2015;11(1):1-15.e4. doi:10.1016/j.jalz.2014.07.003
20. Rowe CC, Jones G, Dore V, et al. Standardized Expression of 18F-NAV4694 and 11C-PiB b-amyloid PET results with the centiloid scale. *J Nucl Med*. 2016;57(8):1233-1237. doi:10.2967/jnumed.115.171595
21. Benedet AL, Leuzy A, Pascoal TA, et al. Stage-specific links between plasma neurofilament light and imaging biomarkers of Alzheimer's disease. *Brain*. 2020;143(12):3793-3804. doi:10.1093/brain/awaa342
22. Salvadó G, Molinuevo JL, Brugulat-Serrat A, et al. Centiloid cut-off values for optimal agreement between

© 2021 Benedet AL et al. *JAMA Neurology*.

PET and CSF core AD biomarkers. *Alzheimers Res Ther*. 2019;11(1):27. doi:10.1186/s13195-019-0478-z
23. Agency EM. ANNEX I SUMMARY OF PRODUCT CHARACTERISTICS. Published 2014. Accessed June 19, 2020. https://www.ema.europa.eu/en/documents/product-information/vizamyl-epar-product-information_en.pdf

© 2021 Benedet AL et al. *JAMA Neurology*.
