# Supplementary material for: Differences Between Plasma and Cerebrospinal Fluid Glial Fibrillary Acidic Protein Levels Across the Alzheimer Disease Continuum
Source: JAMA Neurol. 2021 Oct 18;78(12):1–13. doi: 10.1001/jamaneurol.2021.3671 (PMC8524356; doi:10.1001/jamaneurol.2021.3671)
Supplement: Supplement 2. — Members of the Translational Biomarkers in Aging and Dementia (TRIAD) study, ALFA study, and BioCogBank Paris Lariboisière cohort [file jamaneurol-e213671-s002.pdf]

Supplemental Online Content: Nonauthor Collaborators

*Indicates required information. Only first name, last name, and suffix will appear in PubMed.

***Group Name(s): the Translational Biomarkers in Aging and Dementia (TRIAD) study, Alzheimer's and Families (ALFA) study, and BioCogBank Paris Lariboisière cohort**							
***First Name and Middle Initial(s)**	***Last Name**	***Suffix (eg, Jr, III)**	Academic Degrees	Institution	Location (city, state/province, country)	Role or Contribution, eg, chair, principal investigator	Group (if more than 1 Group listed in the byline) and/or Subgroup (eg, Steering Committee)
Annabella	Beteta			Barcelonaβeta Brain Research Center	Barcelona, Spain		
Raffaele	Cacciaglia			Barcelonaβeta Brain Research Center	Barcelona, Spain		
Alba	Cañas			Barcelonaβeta Brain Research Center	Barcelona, Spain		
Carme	Deulofeu			Barcelonaβeta Brain Research Center	Barcelona, Spain		
Irene	Cumplido			Barcelonaβeta Brain Research Center	Barcelona, Spain		
Ruth	Dominguez			Barcelonaβeta Brain Research Center	Barcelona, Spain		
Maria	Emilio			Barcelonaβeta Brain Research Center	Barcelona, Spain		
Carles	Falcon			Barcelonaβeta Brain Research Center	Barcelona, Spain		
Sherezade	Fuentes			Barcelonaβeta Brain Research Center	Barcelona, Spain		
Laura	Hernandez			Barcelonaβeta Brain Research Center	Barcelona, Spain		
Gema	Huesa			Barcelonaβeta Brain Research Center	Barcelona, Spain		
Jordi	Huguet			Barcelonaβeta Brain Research Center	Barcelona, Spain		
Paula	Marne			Barcelonaβeta Brain Research Center	Barcelona, Spain		
Tania	Menchón			Barcelonaβeta Brain Research Center	Barcelona, Spain		
Grégory	Operto			Barcelonaβeta Brain Research Center	Barcelona, Spain		
Albina	Polo			Barcelonaβeta Brain Research Center	Barcelona, Spain		
Sandra	Pradas			Barcelonaβeta Brain Research Center	Barcelona, Spain		
Anna	Soteras			Barcelonaβeta Brain Research Center	Barcelona, Spain		
Marc	Vilanova			Barcelonaβeta Brain Research Center	Barcelona, Spain		
Natalia	Vilor-Tejedor			Barcelonaβeta Brain Research Center	Barcelona, Spain		
Sinead	Gaubert			Centre de Neurologie Cognitive, GHU APHP Nord Hôpital Lariboisière Fernand-Widal, Paris, France	Paris, France		
Matthieu	Lilamand			Centre de Neurologie Cognitive, GHU APHP Nord Hôpital Lariboisière Fernand-Widal, Paris, France	Paris, France		
Jacques	Hugon			Centre de Neurologie Cognitive, GHU APHP Nord Hôpital Lariboisière Fernand-Widal, Paris, France	Paris, France		
Sandrine	Indart			Centre de Neurologie Cognitive, GHU APHP Nord Hôpital Lariboisière Fernand-Widal, Paris, France	Paris, France		
Alexandra	Fayel			Centre de Neurologie Cognitive, GHU APHP Nord Hôpital Lariboisière Fernand-Widal, Paris, France	Paris, France		
Malika	Gmiz			Centre de Neurologie Cognitive, GHU APHP Nord Hôpital Lariboisière Fernand-Widal, Paris, France	Paris, France		
Hélène	Francisque			Centre de Neurologie Cognitive, GHU APHP Nord Hôpital Lariboisière Fernand-Widal, Paris, France	Paris, France		
Aurélie	Meauzoone			Centre de Neurologie Cognitive, GHU APHP Nord Hôpital Lariboisière Fernand-Widal, Paris, France	Paris, France		

Supplemental Online Content: Nonauthor Collaborators

*Indicates required information. Only first name, last name, and suffix will appear in PubMed.

***First Name and Middle Initial(s)**	***Last Name**	***Suffix (eg, Jr, III)**	Academic Degrees	Institution	Location (city, state/province, country)	Role or Contribution, eg, chair, principal investigator	Group (if more than 1 Group listed in the byline) and/or Subgroup (eg, Steering Committee)
Matthieu	Martinet			Centre de Neurologie Cognitive, GHU APHP Nord Hôpital Lariboisière Fernand-Widal, Paris, France	Paris, France		
Gabrielle	Tence			Centre de Neurologie Cognitive, GHU APHP Nord Hôpital Lariboisière Fernand-Widal, Paris, France			
Mira	Chamoun			Translational Neuroimaging Laboratory, McGill Centre for Studies in Aging, McGill University	Montreal, Canada		
Joseph	Therriault			Translational Neuroimaging Laboratory, McGill Centre for Studies in Aging, McGill University	Montreal, Canada		
Cécile	Tissot			Translational Neuroimaging Laboratory, McGill Centre for Studies in Aging, McGill University	Montreal, Canada		
Gleb	Bezgin			Translational Neuroimaging Laboratory, McGill Centre for Studies in Aging, McGill University	Montreal, Canada		
Serge	Gauthier			Translational Neuroimaging Laboratory, McGill Centre for Studies in Aging, McGill University	Montreal, Canada		
Guilaine	Gagnon			Translational Neuroimaging Laboratory, McGill Centre for Studies in Aging, McGill University	Montreal, Canada		
Alyssa	Stevensson			Translational Neuroimaging Laboratory, McGill Centre for Studies in Aging, McGill University	Montreal, Canada		
